# Comparative effectiveness of acupuncture combined with rehabilitation for post-stroke hemiplegia: a systematic review and network meta-analysis

**DOI:** 10.3389/fneur.2026.1878287

**Published:** 2026-09-29

**Authors:** Hongliang Wang, Xiaoyan Tang, Peizhe Li, Zhihao Dong, Kun Shang, Xin Zhang

**Affiliations:** 1 School of Acupuncture and Tuina, Changchun University of Chinese Medicine, Changchun, Jilin, China; 2 School of Integrated Traditional Chinese and Western Medicine, Changchun University of Chinese Medicine, Changchun, Jilin, China; 3 School of Basic Medical Sciences, Changchun University of Chinese Medicine, Changchun, Jilin, China

**Keywords:** acupuncture, moxibustion, network meta-analysis, post-stroke hemiplegia, rehabilitation, systematic review

## Abstract

**Background:**

Hemiplegia, as a common sequela of stroke, can severely affect patients’ quality of life. Although rehabilitation therapy can improve neurological function and motor abilities, a single therapy often has limited effectiveness in meeting the comprehensive rehabilitation needs. Clinical observations have demonstrated that the integration of acupuncture with rehabilitation therapy yields significant synergistic benefits. However, given the paucity of direct comparative studies between distinct combination therapies, the optimal treatment regimen remains to be definitively established. This study aims to evaluate the efficacy of rehabilitation combined with different acupuncture techniques in treating patients with post-stroke hemiplegia using a network meta-analysis (NMA).

**Methods:**

We systematically searched eight databases for randomized controlled trials (RCTs) related to acupuncture combined with rehabilitation (ACU + RT) for post-stroke hemiplegia, with the searched period from database inception to March 5, 2026. The included literature was analyzed using Bayesian NMA with R software version 4.5.0.

**Results:**

This study included 71 RCTs, involving 6,121 patients and nine interventions. The NMA results indicated that for Fugl-Meyer Assessment (FMA) efficacy, the top three interventions were floating acupuncture combined with rehabilitation (FACU + RT), warm needle moxibustion combined with rehabilitation (WNM + RT), and moxibustion combined with rehabilitation (MOX + RT). However, according to the SUCRA ranking, WNM + RT emerged as the most likely optimal intervention (SUCRA = 76.06%; MD = 16.1, 95%CI: 9.25 to 23.1). Regarding Barthel Index (BI) scores, scalp acupuncture combined with rehabilitation (SA + RT) ranked first (MD = 17.4, 95%CI: 7.47 to 27.1; SUCRA = 86.20%), followed by ACU + RT and MOX + RT. In terms of Modified Barthel Index (MBI) scores, FACU + RT demonstrated the highest efficacy (MD = 16.4, 95%CI: 2.88 to 29.9; SUCRA = 87.87%), followed by MOX + RT and electroacupuncture combined with rehabilitation (EA + RT). Finally, for Modified Ashworth Scale (MAS) scores, SA + RT was ranked first (MD = −1.10, 95%CI: −2.05 to −0.15; SUCRA = 78.38%), with MOX + RT ranking second.

**Conclusion:**

Current evidence indicates that combining different acupuncture methods yields varying therapeutic effects. Regarding the improvement of FMA scores, WNM + RT may be considered the preferred clinical regimen due to the limited evidence supporting FACU + RT (based on a single study). Furthermore, SA + RT proves most effective for improving BI scores and reducing MAS, while FACU + RT exhibits potential benefits for MBI scores. However, as the number of studies reporting MAS data is relatively small (5 cases), the robustness of this result still needs to be further confirmed in future research.

**Systematic review registration:**

CRD420261334951, https://www.crd.york.ac.uk/PROSPERO/view/CRD420261334951.

## 1 Introduction

Stroke, an acute cerebrovascular disorder, results in significant cerebral tissue injury (1). It is not only the second leading cause of death worldwide, but also one of the main factors leading to disability in patients (2). If timely and effective interventions are not implemented, it may result in a variety of lifelong sequelae (3).

Hemiplegia is one of the most common sequela of stroke, and its core feature is a significant impairment of motor function, which is also the key factor leading to the loss of independent living ability in patients (4). Its pathogenesis is complex, frequently stemming from a close association with cerebrovascular disease, traumatic brain injury, intracranial space-occupying lesions, and intracranial infections. These pathological factors may damage the cerebral cortex and related nervous systems, thereby triggering the disease (5). For patients, this not only leads to various functional impairments, thereby reducing the quality of life, but may also trigger psychological disorders, such as anxiety, irritability, or depression (6). For families and society, the necessity of long-term care from relatives and medical teams imposes a significant economic burden and consumes substantial medical resources, constituting an urgent public health issue (7). Therefore, investigating safe, effective, and scalable interventions is of great clinical value and social significance.

At present, the treatment of post-stroke hemiplegia mainly focuses on symptom relief, primarily relying on rehabilitation training and medication therapy (8, 9). Although rehabilitation therapy is crucial for improving the prognosis of hemiplegic patients (10), the uneven development of global medical standards has weakened the capacity of many primary healthcare institutions, making it difficult for them to meet the comprehensive rehabilitation needs of patients (11). Acupuncture, as a core therapy of traditional Chinese medicine, has become an important intervention in the treatment of post-stroke hemiplegia due to its proven efficacy and good safety profile (12, 13).

Evidence indicates that the combination of acupuncture and rehabilitation therapy has a synergistic effect on patients with hemiplegia (14). However, despite the widespread adoption of this combined approach, a critical gap in clinical evidence persists. Most existing studies and meta-analyses have primarily compared combined therapy against rehabilitation alone, lacking direct comparisons among different combined therapies (15). Consequently, it remains unclear which specific combined intervention strategy can bring the greatest clinical advantage. Therefore, this study intends to integrate the existing evidence from randomized controlled trials (RCTs) using network meta-analysis (NMA) to systematically evaluate the clinical efficacy of different acupuncture combined with rehabilitation regimens in the treatment of hemiplegia after stroke. By comparing the relative effects of different combined therapies and identifying the optimal strategy through probability ranking, this study seeks to provide an evidence-based foundation for formulating personalized and precise stroke rehabilitation plans.

## 2 Methods

This study protocol follows the PRISMA guidelines (16) and has been registered in the PROSPERO database (CRD420261334951).

### 2.1 Search strategy

This study systematically searched the following eight databases: PubMed, Cochrane Library, Web of Science, CBM, CNKI, Embase, Wanfang, and VIP Database. The search period was from the establishment of each database to March 5, 2026. The detailed search strategies are shown in Supplementary Table 1.

### 2.2 Inclusion criteria

(1) Patients diagnosed with stroke and exhibiting clear post-stroke hemiplegia or motor dysfunction (recovery stage: 20 days to 6 months post-stroke), regardless of age or gender.
(2) Interventions included various combinations of acupuncture and rehabilitation therapy, such as moxibustion combined with rehabilitation (MOX + RT), abdominal acupuncture combined with rehabilitation (AA + RT), electro-acupuncture combined with rehabilitation (EA + RT), eye acupuncture combined with rehabilitation (EACU + RT), fire needle therapy combined with rehabilitation (FN + RT), scalp acupuncture combined with rehabilitation (SA + RT), warm needle moxibustion combined with rehabilitation (WNM + RT), acupuncture combined with rehabilitation (ACU + RT), and floating acupuncture therapy combined with rehabilitation (FACU + RT).
(3) Control groups received the same rehabilitation treatment protocol as the intervention group, but did not receive any acupuncture intervention.
(4) Each included study reported at least one of the following outcomes. Motor function recovery was quantified using the Fugl-Meyer Assessment (FMA; total score: 226), where higher scores indicate better function (17). The Barthel Index (BI) and Modified Barthel Index (MBI) were used to assess activities of daily living; both range from 0 to 100, with higher scores reflecting greater independence (18). The Modified Ashworth Scale (MAS) assessed muscle tone and spasticity (score range: 0–4), where higher scores denote more severe spasticity (19).
(5) Study designs were limited to RCTs.

### 2.3 Exclusion criteria

(1) Reviews, case reports, conference abstracts, animal studies, or non-RCTs.
(2) Studies with incomplete data or where the full text could not be obtained.
(3) Duplicate publications or reports with overlapping data.
(4) Studies with inconsistent baseline treatments between the two groups.

### 2.4 Selection process

The literature search, screening, and data extraction were independently carried out by Xiaoyan Tang and Zhihao Dong, and were subsequently cross-verified. Duplicate studies were first removed. Subsequently, irrelevant studies were excluded by reviewing the titles and abstracts. Finally, the full texts of the remaining articles were read to determine whether to include them. Any uncertainties or conflicts should be resolved through consultation with Peizhe Li. The following information was extracted: first author, publication year, age, country, disease course, sample size, interventions, treatment duration, outcome indicators, and measurement data.

### 2.5 Risk of bias

The risk of bias assessment was independently conducted by XT and PL using the Cochrane Risk of Bias Assessment Tool 2.0 (RoB 2.0) (20), with cross-validation performed. Any uncertainties or conflicts should be resolved through consultation with ZD.

### 2.6 Statistical analysis

This study selects the Bayesian NMA framework mainly based on its unique advantages in dealing with complex evidence networks. Compared with the frequency-based approach, this framework can provide direct probability inference of therapeutic effects and SUCRA ranking probabilities, making the result interpretation more intuitive. Furthermore, in view of the inherent clinical heterogeneity in acupuncture trials, the Bayesian method can more effectively integrate the evidence and demonstrate stronger robustness to ensure the reliability of the analysis results. The Bayesian NMA was conducted using the gemtc package in R software (version 4.5.0). Outcome measures for statistical analysis were entirely continuous, and effect sizes were expressed as Mean Differences (MD). A statistically significant difference was present if the 95% credible interval (95% CrI) did not include zero. Given the clinical heterogeneity of acupuncture therapy, this study used a random-effects model to account for the potential differences between studies. This NMA used JAGS as the sampler for MCMC simulation, building a total of four independent Markov chains, each of which ran 25,000 iterations. The first 5,000 iterations were used as the warm-up period, and the remaining 20,000 iterations were used for posterior inference. Convergence was assessed using the Potential Scale Reduction Factor and the closer this value was to 1.0, the better the model convergence (21). The relationships between interventions were presented through an evidence network diagram. Since there were no closed loops in this evidence network, an inconsistency model test was not conducted. The comparison results were displayed as a league table. Interventions were ranked according to their surface under the cumulative ranking curve (SUCRA); the closer the SUCRA value is to 100%, the better the effect. A funnel plot was used to assess publication bias for indicators included in more than 10 studies, supplemented by the Egger test.

This study conducted a literature search of previous meta-analyses on post-stroke spasticity and employed the well-established Minimal Clinically Important Difference scores to assess treatment efficacy: FMA > 6 points, BI > 1.85 points, MBI > 5.34 points, and MAS > 1 point (22, 23). Referring to the statistical analysis methods of MAS in the field of stroke rehabilitation through relevant meta-analyses, and considering that MAS has been widely clinically verified and the ordinal meta-analysis method has practical limitations under small sample sizes, this study treated the MAS score as a quasi-continuous variable and analyzed its score using the MD (24, 25).

## 3 Results

### 3.1 Literature screening

A total of 3,079 records were identified through the initial search. After removing duplicates and screening titles and abstracts, 2,923 records were excluded. The full texts of the remaining 156 articles were read, finally, 71 studies were included (Figure 1).

**Figure 1:**
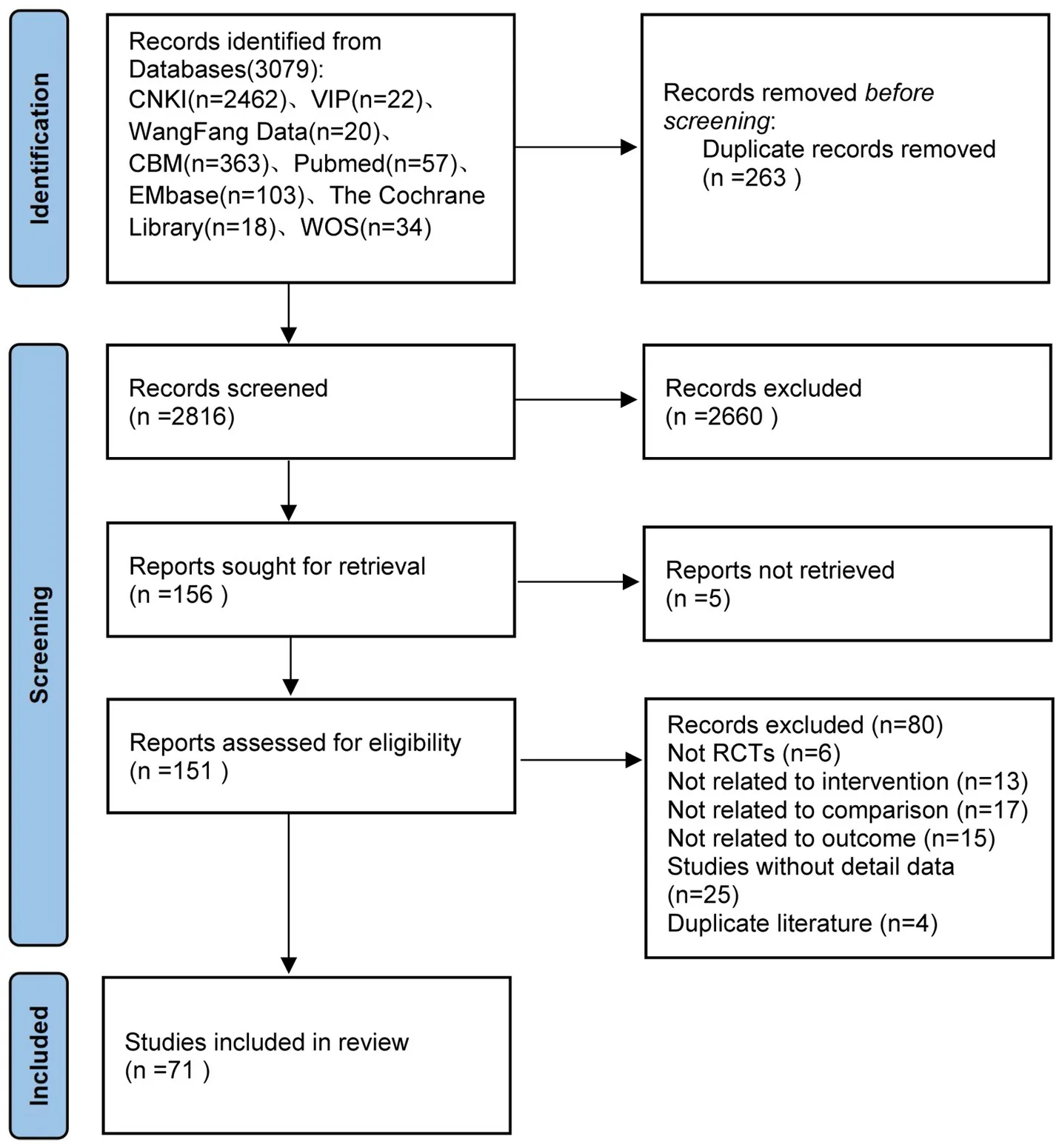
Literature selection flowchart.

### 3.2 Characteristics of the included studies

This analysis included 71 studies (26–96) conducted in China and published between 2008 and 2025. These studies comprised 6,121 patients with post-stroke hemiplegia (intervention group: 3,066, control group: 3,055). Four studies (39, 43, 45, 75) reported patient attrition rates, with the number of dropouts ranging from 1 to 8. Detailed reasons for dropout are provided in Supplementary Table 2. Twenty-two studies (29, 30, 36, 38, 41, 47, 50, 52, 54, 59, 61–63, 71, 76, 80–82, 85, 89, 91, 96) did not report the disease duration. The studies included a total of 9 intervention modalities, namely MOX + RT, FN + RT, SA + RT, WNM + RT, EA + RT, ACU + RT, AA + RT, EACU + RT, and FACU + RT. Study characteristics are presented in Table 1; intervention details are provided in Supplementary Table 3.

**Table 1:** Study characteristics.

Study	Research type	Country	Age (year) (T)	Age (year) (C)	Sample size (T/C)	Treatment (T/C)	Treatment period	Duration (T)	Duration (C)	Outcome indicator
Yang F 2021	RCT	China	57.12 ± 5.12	58.61 ± 4.36	55/55	MOX + RT/RT	4 weeks	31.98 ± 10.15 days	30.75 ± 10.07 days	①②④
Huo XH 2013	RCT	China	59.2 ± 6.31	61.8 ± 8.52	36/32	MOX + RT/RT	20 days	3.87 ± 1.09 weeks	1.14 ± 0.76 weeks	②
Chen ZL 2018	RCT	China	60.25 ± 8.04	59.61 ± 7.03	34/34	MOX + RT/RT	20 days	2.5 ± 0.6 years	2.4 ± 0.3 years	①③④
Xu YM 2013	RCT	China	62.47 ± 9.70	63.10 ± 9.56	30/30	MOX + RT/RT	30 days	/	/	①②④
Wei YX 2016	RCT	China	53.15 ± 14.23	52.52 ± 13.51	44/40	MOX + RT/RT	6 months	/	/	②
Ye Q 2022	RCT	China	62.13 ± 6.79	61.89 ± 7.03	43/43	MOX + RT/RT	8 weeks	10.58 ± 1.26 h	11.01 ± 1.34 h	①②
Xie L 2024	RCT	China	61.29 ± 9.23	63.04 ± 9.94	43/43	EA + RT/RT	84 days	5.87 ± 1.91 months	6.28 ± 1.96 months	①
Feng Z 2017	RCT	China	63 ± 9	67 ± 11	29/30	EA + RT/RT	4 weeks	61.1 ± 52.1 days	67.4 ± 81.7 days	②
Zhou LZ 2011	RCT	China	57 ± 8	58 ± 7	35/35	EA + RT/RT	8 weeks	6.14 ± 1.67 days	6.80 ± 1.84 days	①
Fu Y 2013	RCT	China	63.07 ± 5.12	62.05 ± 6.25	60/60	EA + RT/RT	4 weeks	16.65 ± 2.25 days	13.25 ± 5.25 days	①③
Dai R 2016	RCT	China	66.4 ± 3.2	65.7 ± 4.1	32/31	EA + RT/RT	4 weeks	/	/	①③
Hu DY 2017	RCT	China	64.2 ± 9.1	65.8 ± 8.3	30/30	EA + RT/RT	8 weeks	25.7 ± 5.66 days	26.1 ± 5.31 days	①②
Zhang Y 2023	RCT	China	62.25 ± 4.11	62.41 ± 4.28	38/38	EA + RT/RT	4 weeks	/	/	①②
Fu QH 2016	RCT	China	65.68 ± 8.69	63.80 ± 8.76	56/56	EA + RT/RT	8 weeks	6 months~1 year	6 months~1 year	①②
Zeng HW 2015	RCT	China	65.09 ± 4.57	64.25 ± 4.95	35/36	FN + RT/RT	4 weeks	20 days~6 months	20 days~6 months	①③
Xiao YJ 2016	RCT	China	62.9 ± 5.1	63.1 ± 4.9	30/30	FN + RT/RT	4 weeks	/	/	①
Zhang CX 2023	RCT	China	60.40 ± 3.61	60.34 ± 5.33	40/40	FN + RT/RT	4 weeks	70.50 ± 7.10 days	70.58 ± 6.94 days	①
Xu L 2021	RCT	China	61 ± 1	59 ± 1	33/33	SA + RT/RT	6 weeks	40.8 ± 1.9 days	43.5 ± 1.9 days	①
Zhao GY 2019	RCT	China	60.76 ± 7.43	59.89 ± 8.57	45/45	SA + RT/RT	2 weeks	3.02 ± 0.47 years	2.98 ± 0.57 years	①④
Jun W 2017	RCT	China	62 ± 10	63 ± 8	57/58	SA + RT/RT	4 weeks	2.7 ± 1.6 months	2.3 ± 1.5 months	①③
Wang YJ 2010	RCT	China	63.50 ± 8.46	62.83 ± 8.85	40/40	SA + RT/RT	4 weeks	8.60 ± 4.21 days	9.90 ± 5.50 days	①
Pang L 2022	RCT	China	62.71 ± 9.60	62.68 ± 9.64	40/40	SA + RT/RT	12 weeks	/	/	①③
Zhang WY 2015	RCT	China	66.25 ± 5.90	68.03 ± 4.92	30/30	SA + RT/RT	24 days	38.56 ± 5.12 days	37.96 ± 4.98 days	②
Xu YH 2015	RCT	China	46.3 ± 3.7	45.7 ± 4.8	20/20	SA + RT/RT	4 weeks	6.84 ± 2.36 days	6.86 ± 2.25 days	①③④
Li ZY 2025	RCT	China	60.51 ± 5.62	59.71 ± 7.03	20/20	SA + RT/RT	8 weeks	/	/	①
Hu HY 2018	RCT	China	63.8 ± 5.9	64.9 ± 6.4	56/56	SA + RT/RT	60 days	16.8 ± 5.9 days	15.7 ± 5.8 days	①②
Xiao L 2018	RCT	China	57.14 ± 9.67	57.14 ± 9.67	54/54	SA + RT/RT	12 weeks	/	/	①③
Huang ZJ 2018	RCT	China	65.39 ± 7.34	67.79 ± 9.84	40/40	WNM + RT/RT	4 weeks	4.22 ± 12.10 days	7.91 ± 11.62 days	②
Yin GD 2021	RCT	China	59.24 ± 5.71	58.83 ± 5.46	36/36	WNM + RT/RT	4 weeks	/	/	②
Miao LJ 2023	RCT	China	63.17 ± 10.24	62.89 ± 9.96	42/42	WNM + RT/RT	12 weeks	45.27 ± 12.48 days	44.93 ± 12.76 days	①②
Hu CH 2018	RCT	China	65 ± 15	65 ± 20	76/74	WNM + RT/RT	4 weeks	27.2 ± 8.6 days	28.9 ± 9.5 days	①②
Wu YF 2024	RCT	China	62.15 ± 5.51	61.53 ± 6.12	30/30	WNM + RT/RT	4 weeks	1.98 ± 0.23 months	1.93 ± 0.21 months	②
Li F 2019	RCT	China	58.53 ± 8.41	58.95 ± 8.47	50/50	WNM + RT/RT	4 weeks	27.67 ± 6.04 days	27.35 ± 6.43 days	①②
Han ZX 2018	RCT	China	64.43 ± 10.54	64.97 ± 8.98	30/30	WNM + RT/RT	6 months	/	/	①②
Sun HX 2014	RCT	China	61.15 ± 9.262	60.45 ± 9.467	30/30	ACU + RT/RT	4 weeks	2 ± 1.32 weeks	2 ± 1.02 weeks	①
Huang YP 2020	RCT	China	65.45 ± 4.24	65.53 ± 4.36	48/48	ACU + RT/RT	8 weeks	/	/	①③
Wang XQ 2008	RCT	China	60 ± 11	62 ± 10	40/40	ACU + RT/RT	4 weeks	/	/	①②
Chen XM 2016	RCT	China	62.39 ± 8.94	61.25 ± 8.42	50/50	ACU + RT/RT	4 weeks	/	/	③
Pan RH 2015	RCT	China	61.10 ± 8.95	58.17 ± 9.43	30/30	ACU + RT/RT	4 weeks	2.16 ± 1.19 months	2.27 ± 0.88 months	①③
Wang M 2020	RCT	China	62.33 ± 6.40	50.23 ± 14.35	35/35	ACU + RT/RT	4 weeks	61.54 ± 15.25 days	57.52 ± 15.36 days	①
Luan H 2017	RCT	China	65.32 ± 5.04	64.39 ± 5.30	45/45	ACU + RT/RT	4 weeks	43.68 ± 5.76 days	42.28 ± 6.54 days	①③
Sun K 2010	RCT	China	64.5 ± 9.6	64.7 ± 9.3	45/45	ACU + RT/RT	4 weeks	68.0 ± 32.6 days	75.0 ± 34.2 days	①②
Lang JY 2011	RCT	China	65.9 ± 8.29	63.6 ± 8.73	37/42	ACU + RT/RT	4 weeks	35.4 ± 23.81 days	35.7 ± 20.97 days	①
Lu YQ 2013	RCT	China	61.50 ± 7.32	62.10 ± 8.14	40/40	ACU + RT/RT	2 weeks	35.50 ± 13.72 days	34.90 ± 13.49 days	①
Li ZK 2019	RCT	China	60.32 ± 7.67	61.10 ± 8.02	45/45	ACU + RT/RT	7 days	37.10 ± 13.74 days	35.67 ± 12.49 days	①
Zhou ZH 2011	RCT	China	65 ± 8	65 ± 7	49/45	ACU + RT/RT	4 weeks	/	/	①
Yang HT 2013	RCT	China	64.69 ± 8.90	62.36 ± 9.67	38/38	ACU + RT/RT	2 weeks	34.87 ± 15.92 days	35.06 ± 19.68 days	①
Zhang NX 2010	RCT	China	65.9 ± 11.1	69.2 ± 9.7	40/40	ACU + RT/RT	3 weeks	2.70 ± 1.47 days	3.40 ± 2.34 days	②
Min M 2008	RCT	China	66.24 ± 10.20	65.78 ± 8.46	30/30	ACU + RT/RT	3 months	7.8 ± 1.5 days	8.4 ± 1.6 days	①③
Yingying Z 2021	RCT	China	65.40 ± 11.44	64.88 ± 11.45	40/40	ACU + RT/RT	4 weeks	21.98 ± 6.67 days	22.25 ± 6.26 days	①③
Sun HC 2015	RCT	China	52.7 ± 1.9	51.2 ± 2.4	48/45	ACU + RT/RT	2 weeks	/	/	①②
Chen Y 2018	RCT	China	55 ± 9	55 ± 9	49/47	ACU + RT/RT	8 weeks	15.4 ± 4.9 days	15.3 ± 3.8 days	①③
Chen X 2010	RCT	China	66.7 ± 10.3	67.9 ± 9.4	40/40	ACU + RT/RT	8 weeks	14.5 ± 5.43 days	15.3 ± 5.36 days	①②
Xia QY 2019	RCT	China	56.65 ± 11.20	58.44 ± 11.06	64/64	ACU + RT/RT	12 weeks	63.67 ± 22.12 days	66.21 ± 22.64 days	①③
Wu HP 2022	RCT	China	62.39 ± 9.93	62.34 ± 9.87	51/51	ACU + RT/RT	8 weeks	/	/	①②
Wang HB 2015	RCT	China	56.7 ± 2.5	54.2 ± 3.6	52/52	ACU + RT/RT	2 weeks	/	/	①②
Huang CH 2014	RCT	China	63.2 ± 12.4	63.4 ± 12.2	71/71	ACU + RT/RT	4 weeks	/	/	①③
Guo YP 2016	RCT	China	55.2 ± 3.7	53.7 ± 3.5	58/58	ACU + RT/RT	4 weeks	15.4 ± 6.5 days	12.3 ± 7.4 days	①②
Hao CD 2019	RCT	China	65.7 ± 3.8	64.5 ± 4.1	55/55	ACU + RT/RT	30 days	16.0 ± 1.5 days	15.0 ± 1.9 days	①③
Zheng D 2019	RCT	China	61.3 ± 2.6	61.1 ± 2.4	53/53	ACU + RT/RT	8 weeks	/	/	①
Liu J 2019	RCT	China	41 ± 19	38 ± 25	39/40	ACU + RT/RT	90 days	4.3 ± 2.3 years	4.9 ± 1.7 years	①③
Li LF 2017	RCT	China	66.13 ± 3.1	67.02 ± 3.5	30/30	ACU + RT/RT	4 weeks	9.5 ± 8.5 years	10 ± 9 years	①③
Hu XW 2022	RCT	China	60.98 ± 4.22	61.25 ± 1.41	35/35	ACU + RT/RT	4 weeks	17.12 ± 3.08 days	16.85 ± 3.02 days	①②
Wang QW 2014	RCT	China	63.2 ± 7.9	65.2 ± 8.9	30/30	AA+RT/RT	8 weeks	≤1 month	≤1 month	①③
Jin LQ 2018	RCT	China	56.43 ± 9.84	58.60 ± 7.33	60/60	AA+RT/RT	8 weeks	9.0 ± 1.9 weeks	10.0 ± 1.7 weeks	①
Wang CX 2015	RCT	China	66.3 ± 6.1	65.5 ± 7.6	40/40	AA+RT/RT	8 weeks	/	/	①②
Wang KX 2023	RCT	China	72.09 ± 3.43	71.40 ± 3.48	65/65	EACU+RT/RT	4 weeks	3.15 ± 0.78	2.98 ± 0.67 months	①③
Dong Y 2021	RCT	China	65.02 ± 6.58	63.13 ± 5.57	53/53	EACU+RT/RT	4 weeks	4.30 ± 0.72 days	4.24 ± 0.76 days	①
Sun Y 2021	RCT	China	57.51 ± 9.09	58.22 ± 8.78	56/56	EACU+RT/RT	4 weeks	3.28 ± 0.85 months	3.03 ± 0.93 months	①②
Zou XM 2021	RCT	China	59.34 ± 6.63	59.11 ± 6.51	46/46	EACU+RT/RT	4 weeks	33.20 ± 3.37 days	33.26 ± 3.42 days	①
Huang SZ 2016	RCT	China	57.32 ± 7.14	58.46 ± 5.97	60/60	FACU+RT/RT	8 weeks	/	/	①③

MOX + RT, moxibustion combined with rehabilitation; EA + RT, electroacupuncture combined with rehabilitation; FN + RT, fire needle combined with rehabilitation; SA + RT, scalp acupuncture combined with rehabilitation; WNM + RT, warm needle moxibustion combined with rehabilitation; ACU + RT, acupuncture combined with rehabilitation; AA + RT, abdominal acupuncture combined with rehabilitation; EACU + RT, eye acupuncture combined with rehabilitation; FACU + RT, floating acupuncture combined with rehabilitation; RT, rehabilitation. ①, Fugl-Meyer Assessment (FMA) scores; ②, Barthel Index (BI) scores; ③, Modified Barthel Index (MBI) scores; ④, Modified Ashworth Scale (MAS) scores.

### 3.3 Risk of bias

Among the 71 included studies, four studies (53, 56, 58, 68) (5.63%) were rated as having a high risk of bias, 20 studies (26, 32, 35, 43, 44, 50–52, 55, 57, 64, 66, 67, 69, 70, 73, 77, 78, 80, 84) (28.17%) were rated as having some concerns. The remaining 47 studies (27–31, 33, 34, 36–42, 45–49, 54, 59–65, 71, 72, 74–76, 79, 81–83, 85–96) (66.20%) were evaluated as having a low risk of bias. For detailed assessment dimensions, see Supplementary Figures 1, 2. Only 30 studies (26–28, 30–33, 35–39, 42, 44, 45, 47, 48, 50, 52–54, 59, 63, 64, 67–69, 72, 74, 77) described the randomization process in detail, while the remaining studies did not fully explain it. Coupled with the unique characteristics of acupuncture interventions, which make blinding difficult, the risk of bias might be increased.

### 3.4 Network meta-analysis

#### 3.4.1 FMA

Sixty-two articles (26, 28, 29, 31, 32, 34–47, 49–52, 55, 56, 58–62, 64–72, 74–96) reported changes in FMA scores, involving a total of 5,458 patients. Convergence diagnostics indicated that the model converged well (see Supplementary Figure 3). The network evidence diagram included nine interventions: MOX + RT, EA + RT, FN + RT, SA + RT, WNM + RT, ACU + RT, AA + RT, EACU + RT, and FACU + RT (see Figure 2 and Supplementary Table 4).

**Figure 2:**
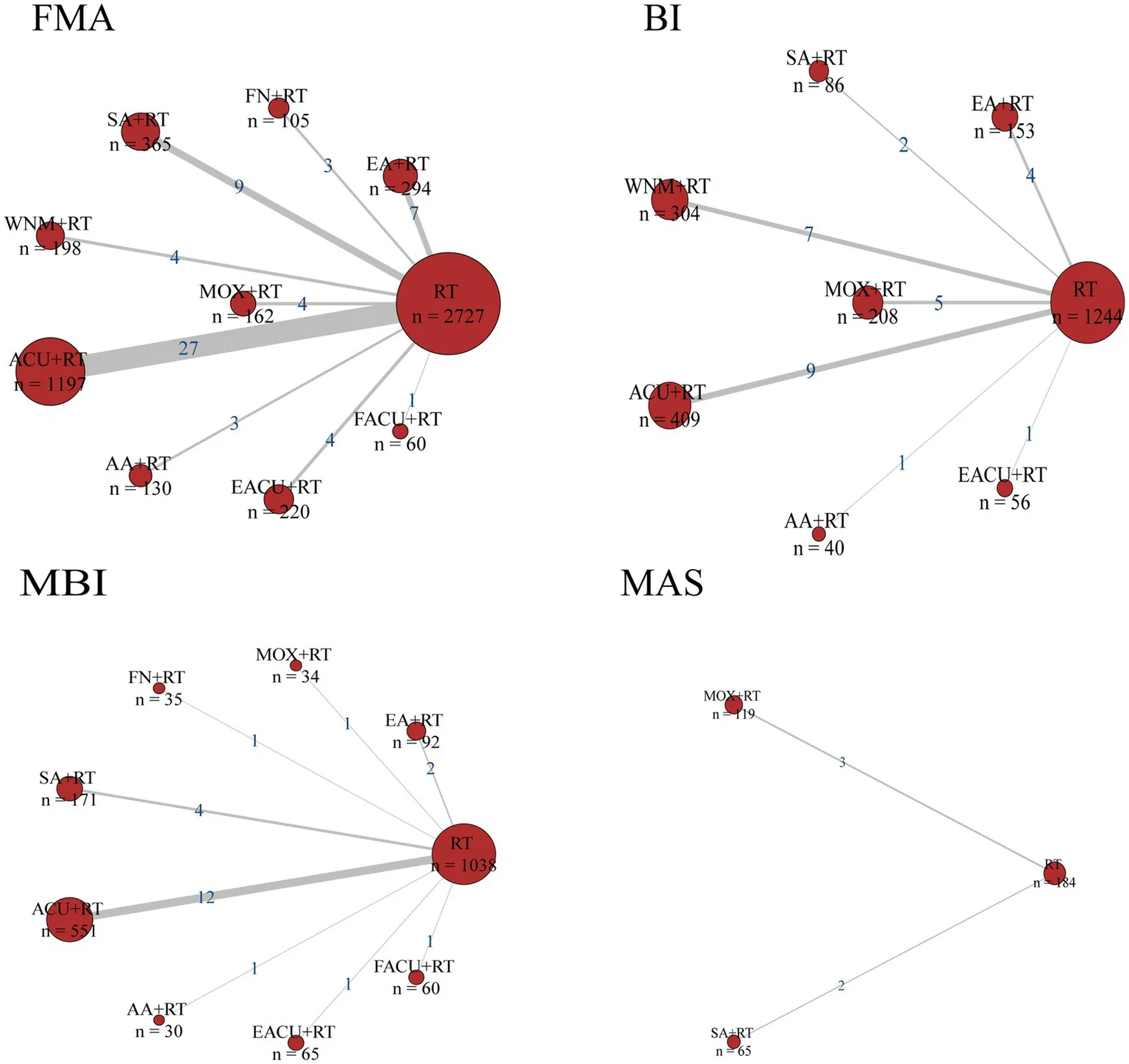
Network plot of evidence. The width of the lines represents the number of studies between each pair of interventions, and the size of the nodes reflects the cumulative sample size for each intervention. MOX + RT, moxibustion combined with rehabilitation; EA + RT, electroacupuncture combined with rehabilitation; FN + RT, fire needle combined with rehabilitation; SA + RT, scalp acupuncture combined with rehabilitation; WNM + RT, warm needle moxibustion combined with rehabilitation; ACU + RT, acupuncture combined with rehabilitation; AA + RT, abdominal acupuncture combined with rehabilitation; EACU + RT, eye acupuncture combined with rehabilitation; FACU + RT, floating acupuncture combined with rehabilitation.

Compared with the control group, 8 combination therapies were effective in improving FMA scores among patients with post-stroke hemiplegia. Among these, FACU + RT (MD = 16.4, 95% CrI: 2.88 to 29.9) and WNM + RT (MD = 16.1, 95% CrI: 9.25 to 23.1) demonstrated particularly favorable efficacy. Following these, MOX + RT (MD = 15.7, 95% CrI: 8.57 to 22.8), SA + RT (MD = 14.3, 95% CrI: 9.62 to 19.0), FN + RT (MD = 13.4, 95% CrI: 5.43 to 21.4), EACU + RT (MD = 13.3, 95% CrI: 6.61 to 20.1), ACU + RT (MD = 12.5, 95% CrI: 9.77 to 15.2), and EA + RT (MD = 10.4, 95% CrI: 5.29 to 15.5) also exhibited positive efficacy (see Table 2 and Supplementary Figure 4).

**Table 2:** League table of intervention comparisons.

Treatment	BI									
FMA	A	3.2 (−6.44, 12.86)	-	−5.59 (−16.99, 6.1)	2.71 (−5.25, 10.61)	−2.53 (−10.16, 5.14)	6.86 (−7.58, 21.49)	−0.88 (−17.23, 15.57)	–	**11.85 (5.85, 17.9)**
	5.25 (−3.47, 13.99)	B	-	−8.75 (−20.97, 3.72)	−0.47 (−9.58, 8.72)	−5.72 (−14.64, 3.19)	3.66 (−11.47, 18.86)	−4.06 (−21.01, 13.11)	–	**8.64 (1.11, 16.2)**
	2.26 (−8.46, 12.92)	−2.98 (−12.5, 6.52)	C	–	–	–	–	–	–	–
	1.29 (−7.15, 9.86)	−3.94 (−10.83, 3.06)	−0.96 (−10.19, 8.32)	D	8.31 (−2.95, 19.22)	3.04 (−7.98, 13.81)	12.41 (−4.14, 28.87)	4.72 (−13.56, 22.76)	–	**17.42 (7.47, 27.14)**
	−0.47 (−10.47, 9.49)	−5.72 (−14.45, 2.92)	−2.73 (−13.4, 7.83)	−1.77 (−10.28, 6.52)	E	−5.25 (−12.29, 1.77)	4.15 (−9.99, 18.35)	−3.6 (−19.77, 12.58)	–	**9.13 (3.99, 14.27)**
	3.19 (−4.39, 10.82)	−2.06 (−7.83, 3.73)	0.93 (−7.55, 9.4)	1.88 (−3.55, 7.25)	3.66 (−3.71, 11.18)	F	9.39 (−4.56, 23.44)	1.66 (−14.35, 17.68)	–	**14.4 (9.68, 19.14)**
	**15.52 (5.27, 25.88)**	**10.26 (1.24, 19.31)**	**13.25 (2.36, 24.2)**	**14.2 (5.4, 22.98)**	**15.99 (5.85, 26.25)**	**12.36 (4.35, 20.25)**	G	−7.73 (−27.73, 12.39)	–	5 (−8.19, 18.18)
	2.31 (−7.39, 12.14)	−2.94 (−11.4, 5.56)	0.06 (−10.39, 10.54)	1.01 (−7.2, 9.19)	2.77 (−6.89, 12.51)	−0.89 (−8.09, 6.39)	−13.2 (−23.22, −3.16)	H	–	12.75 (−2.66, 28.06)
	−0.67 (−15.85, 14.43)	−5.92 (−20.36, 8.49)	−2.94 (−18.51, 12.68)	−1.98 (−16.37, 12.14)	−0.2 (−15.45, 14.86)	−3.87 (−17.69, 9.87)	−16.2 (−31.63, −0.76)	−3.02 (−18.14, 12.1)	I	–
	**15.66 (8.57, 22.78)**	**10.41 (5.29, 15.51)**	**13.4 (5.43, 21.4)**	**14.33 (9.62, 19)**	**16.11 (9.25, 23.13)**	**12.47 (9.77, 15.15)**	0.15 (−7.32, 7.62)	**13.35 (6.61, 20.05)**	**16.35 (2.88, 29.88)**	J

The upper-right triangle corresponds to BI outcomes (row vs. column), while the lower-left triangle corresponds to FMA outcomes (column vs. row). Statistically significant results are highlighted in bold. A, moxibustion combined with rehabilitation; B, electroacupuncture combined with rehabilitation; C, fire needle combined with rehabilitation; D, scalp acupuncture combined with rehabilitation; E, warm needle moxibustion combined with rehabilitation; F, acupuncture combined with rehabilitation; G, abdominal acupuncture combined with rehabilitation; H, eye acupuncture combined with rehabilitation; I, floating acupuncture combined with rehabilitation; J, rehabilitation.

The SUCRA ranking results (see Table 3) indicated: WNM + RT (76.06%) > MOX + RT (73.03%) > FACU + RT (71.56%) > SA + RT (65.61%) > FN + RT (58.07%) > EACU + RT (57.69%) > ACU + RT (49.72%) > EA + RT (36.44%) > AA+RT (6.34%) > RT (5.49%). The cumulative probability plot revealed that WNM + RT had the largest area under the curve (see Figure 3), indicating that it was the most reliable intervention in improving FMA scores. The FMA evidence summary table is provided in Supplementary Table 5.

**Table 3:** SUCRA rankings of interventions for different outcomes.

Rank	FMA	SUCRA (%)	BI	SUCRA (%)	MBI	SUCRA (%)	MAS	SUCRA (%)
1	WNM + RT	76.06	SA + RT	86.20	FACU+RT	87.87	SA + RT	78.38
2	MOX + RT	73.03	ACU + RT	76.68	MOX + RT	81.65	MOX + RT	70.29
3	FACU+RT	71.56	EACU+RT	62.12	EA + RT	63.60	RT	1.33
4	SA + RT	65.61	MOX + RT	60.22	ACU + RT	60.17	–	–
5	FN + RT	58.07	WNM + RT	42.43	AA+RT	50.38	–	–
6	EACU+RT	57.69	EA + RT	41.12	FN + RT	42.69	–	–
7	ACU + RT	49.72	AA+RT	27.20	EACU+RT	34.04	–	–
8	EA + RT	36.44	RT	4.04	SA + RT	27.13	–	–
9	AA+RT	6.34	-	-	RT	2.47	–	–
10	RT	5.49	-	-	-	-	–	–

MOX+RT, moxibustion combined with rehabilitation; EA+RT, electroacupuncture combined with rehabilitation; FN+RT, fire needle combined with rehabilitation; SA+RT, scalp acupuncture combined with rehabilitation; WNM+RT, warm needle moxibustion combined with rehabilitation; ACU+RT, acupuncture combined with rehabilitation; AA+RT, abdominal acupuncture combined with rehabilitation; EACU+RT, eye acupuncture combined with rehabilitation; FACU+RT, floating acupuncture combined with rehabilitation.

**Figure 3:**
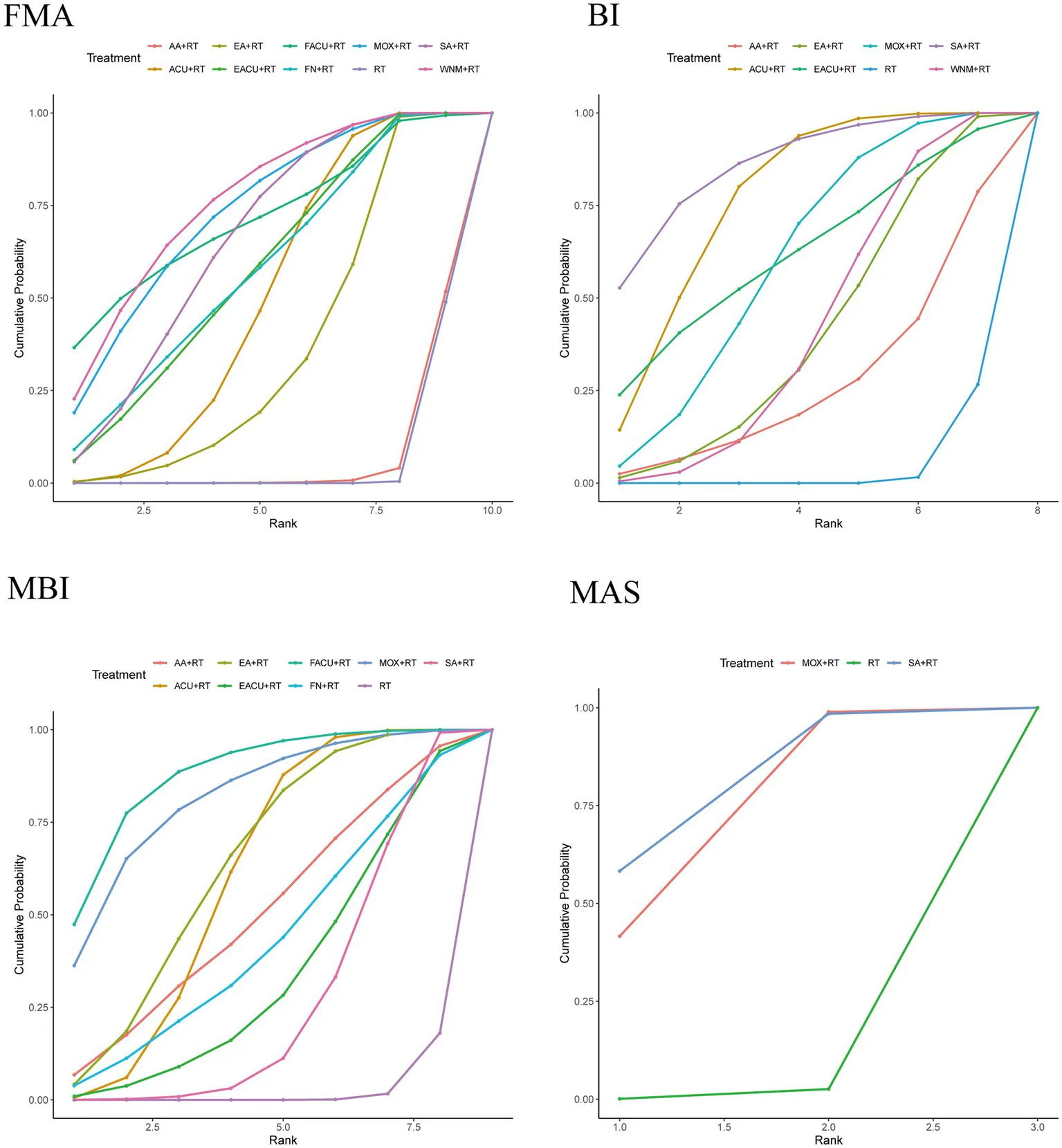
Cumulative probability plot. MOX + RT, moxibustion + rehabilitation; EA + RT, electroacupuncture + rehabilitation; FN + RT, fire needle + rehabilitation; SA + RT, scalp acupuncture + rehabilitation; WNM + RT, warm needle moxibustion + rehabilitation; ACU + RT, acupuncture + rehabilitation; AA+RT, abdominal acupuncture + rehabilitation; EACU + RT, eye acupuncture + rehabilitation; FACU + RT, floating acupuncture + rehabilitation.

#### 3.4.2 BI

A total of 29 articles (26, 27, 29–31, 33, 37–39, 48, 51, 53–59, 62, 67, 73, 76, 78, 80, 81, 83, 88, 91, 94) reported changes in BI scores from pre-treatment to post-treatment, involving 2,500 patients. Convergence diagnostics showed that the model converged well (see Supplementary Figure 5). The network evidence diagram included 7 interventions: SA + RT, MOX + RT, EA + RT, WNM + RT, ACU + RT, AA + RT, and EACU + RT (see Figure 2 and Supplementary Table 4).

Compared with the control group, seven combination therapies were effective. Among these, SA + RT (MD = 17.4, 95% CrI: 7.47 to 27.1) demonstrated a significant advantage. Following these, ACU + RT (MD = 14.4, 95% CrI: 9.68, 19.1), MOX + RT (MD = 11.8, 95% CrI: 5.85 to 17.9), WNM + RT (MD = 9.13, 95% CrI: 3.99 to 14.3), and EA + RT (MD = 8.64, 95% CrI: 1.11 to 16.2) also exhibited positive efficacy (see Table 2 and Supplementary Figure 6).

The SUCRA ranking results (see Table 3) indicated: SA + RT (86.20%) > ACU + RT (76.68%) > EACU + RT (62.12%) > MOX + RT (60.22%) > WNM + RT (42.43%) > EA + RT (41.12%) > AA + RT (27.20%) > RT (4.04%). The cumulative probability plot showed that SA + RT had the greatest area under the curve (see Figure 3), suggesting that SA + RT was more effective than other interventions in improving BI scores. The BI evidence summary table is provided in Supplementary Table 6.

#### 3.4.3 MBI

A total of 23 articles (28, 35, 36, 40, 45, 47, 49, 52, 61, 63, 64, 66, 74, 75, 77, 79, 82, 84, 86, 87, 89, 92, 96) documented changes in MBI scores from pre-treatment to post-treatment, involving 2,076 participants. Convergence diagnostics indicated that the model converged well (see Supplementary Figure 7). The network evidence diagram included 8 interventions: MOX + RT, FN + RT, EA + RT, ACU + RT, AA + RT, EACU + RT, SA + RT, and FACU + RT (see Figure 2 and Supplementary Table 4).

Compared with the control group, five combination therapies were effective. Among them, FACU + RT (MD = 21.1, 95% CrI: 10.2 to 32.1) demonstrated a significant advantage. Following these, MOX + RT (MD = 19.7, 95% CrI: 6.72 to 32.3), EA + RT (MD = 14.4, 95% CrI: 6.80 to 22.1), ACU + RT (MD = 13.7, 95% CrI: 10.2 to 17.0), and SA + RT (MD = 7.06, 95% CrI: 1.22 to 12.9) also exhibited positive efficacy (see Table 4 and Supplementary Figure 8).

**Table 4:** League table of intervention comparisons.

Treatment	MAS								
MBI	A	–	–	0.09 (−1.11, 1.32)	–	–	–	–	−1.01 (−1.75, −0.23)
	5.26 (−9.8, 20.04)	B	–	–	–	–	–	–	–
	9.72 (−9.14, 28.28)	4.47 (−11.26, 20.12)	C	–	–	–	–	–	–
	12.61 (−1.57, 26.51)	7.35 (−2.31, 16.98)	2.88 (−11.9, 17.82)	D	–	–	–	–	−1.1 (−2.05, −0.15)
	6.01 (−7.26, 19.17)	0.75 (−7.52, 9.22)	−3.72 (−17.69, 10.46)	−6.61 (−13.26, 0.21)	F	–	–	–	–
	7.92 (−11.24, 26.82)	2.64 (−13.37, 18.58)	−1.82 (−21.25, 17.77)	−4.72 (−19.84, 10.48)	1.9 (−12.58, 16.19)	G	–	–	–
	11.51 (−5.49, 28.18)	6.22 (−7.11, 19.48)	1.75 (−15.78, 19.2)	−1.12 (−13.44, 11.09)	5.44 (−6, 16.64)	3.59 (−14.37, 21.23)	H	–	–
	−1.42 (−18.33, 15.24)	−6.67 (−20.01, 6.6)	−11.16 (−28.66, 6.45)	−14.04 (−26.45, −1.69)	−7.42 (−19.01, 3.86)	−9.35 (−27.25, 8.48)	−12.9 (−28.2, 2.46)	I	–
	19.66 (6.72, 32.33)	14.4 (6.8, 22.09)	9.94 (−3.68, 23.63)	7.06 (1.22, 12.93)	13.66 (10.24, 16.96)	11.75 (−2.25, 25.81)	8.21 (−2.53, 19.02)	21.08 (10.23, 32.09)	J

The upper-right triangle corresponds to MAS outcomes (row vs. column), while the lower-left triangle corresponds to MBI outcomes (column vs. row). Statistically significant results are highlighted in bold. A, moxibustion combined with rehabilitation; B, electroacupuncture combined with rehabilitation; C, fire needle combined with rehabilitation; D, scalp acupuncture combined with rehabilitation; F, acupuncture combined with rehabilitation; G, abdominal acupuncture combined with rehabilitation; H, eye acupuncture combined with rehabilitation; I, floating acupuncture combined with rehabilitation; J, rehabilitation.

The SUCRA ranking results (see Table 3) indicated: FACU + RT (87.87%) > MOX + RT (81.65%) > EA + RT (63.60%) > ACU + RT (60.17%) > AA+RT (50.38%) > FN + RT (42.69%) > EACU + RT (34.04%) > SA + RT (27.13%) > RT (2.47%). The cumulative probability plot presented that FACU + RT had the greatest area under the curve (see Figure 3), suggesting that FACU + RT was more effective than other types in improving MBI scores. However, given its weak evidence base (based on only one study), the outcomes should be interpreted with caution. The MBI evidence summary table is provided in Supplementary Table 7.

#### 3.4.4 MAS

A total of 5 articles (26, 28, 29, 44, 49) reported changes in MAS scores from pre-treatment to post-treatment, involving 368 participants. Convergence diagnostics indicated that the model converged well (see Supplementary Figure 9). The network evidence diagram included two interventions: MOX + RT and SA + RT (see Figure 2 and Supplementary Table 4).

Compared with the RT group, both combination therapies were effective in reducing the MAS scores of patients with post-stroke hemiplegia. SA + RT (MD = -1.10, 95% CrI: −2.05 to −0.153) showed the greatest improvement. MOX + RT (MD = -1.01, 95% CrI: −1.75 to −0.229) also exhibited positive efficacy. (see Table 4 and Supplementary Figure 10).

The SUCRA ranking results (see Table 3) indicated: SA + RT (78.38%) > MOX + RT (70.29%) > RT (1.33%). The cumulative probability plot showed that the area under the curve for SA + RT was the largest (see Figure 3), indicating that it was more effective than other treatments in reducing MAS scores. Furthermore, since there is limited research on MAS data, the conclusions regarding spasticity relief remain preliminary. Although the data suggest a positive trend, given the small sample size, the robustness of these findings requires further confirmation in future studies. The MAS evidence summary table is provided in Supplementary Table 8.

### 3.5 Publication bias

The funnel plot (Supplementary Figure 11) showed a symmetrical shape overall, with the majority of observations distributed symmetrically around the central line. The results of Egger’s linear regression test showed that the differences in FMA (*p* = 0.221), BI (*p* = 0.179), and MBI (*p* = 0.377) were not statistically significant, indicating that the likelihood of publication bias in this study was low. However, it should be noted that the number of studies on certain interventions (such as FACU + RT) was limited, which might affect the reliability of publication bias analysis. Detailed results are provided in Supplementary Table 9.

### 3.6 Adverse effect

Among the 71 included studies, only four (32, 43, 71, 78) recorded adverse events (AEs), with a reported rate of 5.6%. Xie et al. (32) observed that there were six AEs in the EA + RT group, while there were four in the RT-only group. The main adverse reactions (ADRs) included dizziness, nausea, and localized tingling, but no statistically significant differences were found between the two groups (*p* > 0.05). Xu et al. (43) observed one case of local hematoma at the acupuncture site and one case of back muscle soreness, with no serious ADRs occurring. The other two studies (71, 78) reported that no AEs occurred during the treatment. Existing limited data indicated that no serious AEs had been observed with combined acupuncture treatment, but it should be noted that the vast majority of studies had not adequately recorded and reported safety data. Therefore, the existing evidence was still insufficient to support a definitive conclusion. More high-quality studies are needed in the future to verify this conclusion.

## 4 Discussion

### 4.1 Summary of main findings

This study used a NMA to systematically evaluate the effects of nine interventions on patients with post-stroke hemiplegia. Considering the effect size and SUCRA ranking: In terms of improving FMA scores, FACU + RT showed the largest effect size (MD = 16.4); however, since this intervention was included in only one study, the confidence interval was relatively wide (95% CI: 2.88 to 29.9), resulting in considerable uncertainty in the results. In contrast, WNM + RT, despite having a slightly lower point estimate (MD = 16.1), exhibited a narrower confidence interval (95% CrI: 9.25 to 23.1), resulting in more robust findings and thus ranking highest in the SUCRA analysis (76.06%). Additionally, SA + RT demonstrated the best performance in enhancing BI scores (SUCRA = 86.20%, MD = 17.4, 95% CrI: 7.47 to 27.1) and reducing MAS scores (SUCRA = 78.38%, MD = −1.10, 95% CrI: −2.05 to −0.153). Moreover, FACU + RT showed the greatest potential efficacy in improving MBI scores (SUCRA = 87.87%, MD = 21.1, 95% CrI: 10.2 to 32.1), suggesting that different acupuncture-combined regimens may exhibit varying efficacies across different dimensions of functional recovery in stroke patients with hemiplegia.

### 4.2 Potential mechanisms

Acupuncture combination therapies exhibit different effects on various dimensions of stroke-induced hemiplegia, which may be attributable to the underlying mechanisms of each acupuncture technique.

(1) WNM can directly stimulate the central nervous system, promoting the repair of damaged neurons (97). Meanwhile, it can also enhance local blood circulation in the affected limb, relieve muscle spasm, and create favorable conditions for the recovery of function (98). First, Acupuncture stimulates the nervous system, upregulating BDNF and TrkB expression. This step activates the PI3K/Akt pathway along with MAPK/ERK signaling, promoting synaptic remodeling and neural circuit reconstruction to maintain neuronal integrity (99, 100). Second, acupuncture can significantly reduce the expression of Nogo-A as well as its receptor NgR, thereby relieving the inhibition of axonal growth (101). Concurrently, it increases the expression of nerve growth factor (NGF). Through the activation of the TrkA receptor, NGF stimulates the PLC-γ and MAPK pathways, promoting axonal sprouting and myelin formation. Ultimately, it achieves the restoration and reconstruction of neural function (102). On the other hand, acupuncture can inhibit the activation of inflammatory cells and the release of pro-inflammatory cytokines, thereby balancing the inflammatory microenvironment and reducing secondary nerve damage (103). Furthermore, it regulates vascular smooth muscle cell function, improving blood supply to ischemic areas and penumbral zones, thereby providing adequate nutritional support for neural repair following stroke (104). Moreover, the heat of moxibustion is transmitted along the needle shaft into the deep tissues, not only effectively improves local microcirculation but also further enhances the efficiency of neuroplasticity at a systemic level (105), ultimately leading to improvements in motor function and activities of daily living.
(2) SA can directly stimulate the cortical areas of the brain corresponding to the hemiplegic limbs, increasing the excitability of neurons (106). It can also block muscle spasms at the molecular level by regulating neurotransmitters (107). Studies have shown that SA can not only improve blood supply to the ischemic penumbra (108), but also effectively relieve muscle spasms through inhibitory regulation of spinal KCC2 mediated by activation of 5-HT₂A receptors (109). From a neurological perspective, SA can enhance the excitability of cortical neurons, increase the sensitivity of hemiplegic areas to various stimuli, and thereby improve the coordination between agonist, antagonist, and synergistic muscles (110).
(3) FACU is a modern acupuncture technique. By fanwise sweeping the loose connective tissue beneath the skin and combining with passive movements of the hemiplegic limbs, it can release adhered muscles and fascia, improve circulation, enhance metabolism, accelerate the absorption of inflammation, thereby reducing tissue edema and promoting repair (111, 112). In terms of neural regulation, FACU can increase the contact area with subcutaneous nerve endings. This promotes the activation of opioid receptors and the release of endogenous analgesic substances, thereby improving pain-related motor dysfunction (113). From a biological perspective, the mechanical tension exerted by FACU on connective tissue can trigger a piezoelectric effect. This effect will change the state of ion channels on the cell membrane, thereby relieving muscle spasms and promoting the recovery of motor function (114).

### 4.3 Comparability of scales

Both BI and MBI can be used to assess activities of daily living, but there are essential differences in the granularity of their scores. Combined analysis may introduce significant heterogeneity bias. BI uses a coarse granularity score of 2 to 4 levels (such as 0/5/10/15), which cannot distinguish the subtle differences in functional states such as “requiring supervision” and “requiring full assistance”. MBI adopts a 5-level fine granularity score (such as 0/2/5/8/10), which can precisely capture the changes in independent capabilities by increasing the intermediate score. If the two are combined, the highly sensitive data of MBI will be diluted by the coarse-grained data of BI, ultimately leading to distorted effect size estimation.

Although BI and MBI were not combined for analysis, the results of studies within the same scale were comparable. All the included BI studies adopted the “Nursing Grading” health industry standard WS/T 431–2013, with a total score of 100 points, divided into three grades: good (>60 points), medium (41–60 points), and poor (≤40 points). The scoring grades were completely consistent with the grading boundaries, and the MD could be directly combined. All the included MBI studies adopted Shah’s modified version, with a total score of 100 points. The 5-level scores correspond to independent ability levels of 0, 20, 50, 80, and 100%, respectively. The specific scores were calculated based on the weighted item weights (for example, the bathing item was 0/3/8/12/15, and the toilet item was 0/2/5/8/10). The 5-level scoring criteria and the logic of score assignment are unified, and the Minimal Clinically Important Difference (5–10 points) of MBI has been widely verified in the field of stroke rehabilitation, and the results are clinically comparable.

### 4.4 Heterogeneity and standardization challenges

The interpretation of the results of this study needs to carefully consider the heterogeneity existing in the implementation process of the intervention measures. Firstly, regarding the “conventional acupuncture” of the experimental group, although all the included studies were based on the theory of meridians and acupoints, the specific operations (such as the depth of needle insertion, the technique of needle movement, the duration of needle retention, and the parameters of electroacupuncture, etc.) showed certain differences among different studies. This to some extent reflects the clinical characteristics of individualized acupuncture diagnosis and treatment, but it also means that the intervention measures in different studies are not completely homogeneous, which may introduce certain clinical heterogeneity.

Secondly, a completely unified standard plan for the “conventional rehabilitation” of the control group has not yet been formed. The rehabilitation strategies included in the study covered a variety of combinations such as passive joint movement, side turning exercises, sit-stand training, and walking training. In addition, the intensity of intervention (frequency and duration of each session) also showed a certain degree of diversity among various studies. Due to the level of detail of the original research report, we cannot completely rule out the possibility that the differences in the intensity of rehabilitation training, as potential confounding factors, had a certain impact on the final functional outcome.

Therefore, in the future, efforts should be made to establish more rigorous acupuncture operation norms (refer to the STRICTA statement), and quantitatively describe the details of rehabilitation intervention to minimize heterogeneous interference, thereby more accurately evaluating the clinical efficacy of different intervention measures.

### 4.5 Study limitations

Although the results of this study provide some insights into the treatment of post-stroke hemiplegia, there are still the following limitations.

(1) The limited number of studies investigating certain interventions, such as FACU + RT, may compromise the precision of the results. Consequently, these interventions carry less weight in the NMA, and their SUCRA rankings lack the robustness to reliably support the advantages suggested by the observed effect sizes.
(2) There are certain limitations regarding the characteristics of included interventions and the quality of the literature. Although we used the ROB 2.0 tool to assess quality, the inherent difficulty of implementing double-blind designs in acupuncture trials, compounded by insufficient descriptions of randomization and allocation concealment in some studies, resulted in only 66.20% of the studies being rated as having a low risk of bias. Such limitations may introduce potential bias into the study results.
(3) This study conducted a statistical analysis of MAS data using the MD, but this method may not be able to fully capture the inherent nonlinear characteristics and ordinal properties in spasm assessment. Furthermore, given that only five studies reporting MAS outcomes were included, this makes the evidence base supporting the conclusion of “spasticity reduction” rather limited.
(4) Due to the limited number of RCTs directly comparing the interventions, this study primarily relies on indirect comparison methods. This issue may lead to uncertainty in the precision of effect estimates, thereby reducing the level of evidence for the conclusion.
(5) The scope of the study is relatively limited. As all included studies were conducted in China, the applicability of the findings to other countries is limited. Future validation requires the implementation of more multicenter clinical trials. Meanwhile, repetitive studies from different countries are crucial for clarifying the applicable value of this result in various medical contexts.

## 5 Conclusion

Current evidence suggests that different acupuncture combination therapies exhibit differential advantages in promoting multidimensional functional recovery among stroke patients with hemiplegia. Regarding FMA scores, although the effect size of WNM + RT (MD = 16.1) is marginally lower than that of FACU + RT (MD = 16.4), it remains the preferred clinical option given the greater robustness of its results. Additionally, SA + RT exhibits significant advantages in reducing MAS scores and improving BI scores, while FACU + RT shows potential efficacy in enhancing MBI scores. However, given that the number of studies included in the MAS data is relatively small (5 cases) and the overall quality of the studies is relatively low, the above conclusion still needs to be further verified through more high-quality RCTs.

## Data Availability

The original contributions presented in the study are included in the article/Supplementary material, further inquiries can be directed to the corresponding authors.

## References

[1] Herpich F Rincon F. Management of Acute Ischemic Stroke. Crit Care Med. (2020) 48:1654–63. doi: 10.1097/CCM.0000000000004597, 32947473 PMC7540624

[2] Prabhakaran S Ruff I Bernstein RA. Acute stroke intervention: a systematic review. JAMA. (2015) 313:1451–62. doi: 10.1001/jama.2015.3058, 25871671

[3] Barthels D Das H. Current advances in ischemic stroke research and therapies. Biochim Biophys Acta Mol basis Dis. (2020) 1866:165260. doi: 10.1016/j.bbadis.2018.09.012, 31699365 PMC6981280

[4] Chandel V Sinha A Ghose A. "Towards Accessible & Affordable Home-Rehabilitation for Stroke Patients". In: Annu Int Conf IEEE Eng Med Biol Soc, Ed. Sunghoon Lee (2025). p. 1–7. 10.1109/EMBC58623.2025.11254338 41336727

[5] Tan KS Pandian JD Liu L Toyoda K Leung TWH Uchiyama S . Stroke in Asia. Cerebrovasc Dis Extra. (2024) 14:58–75. doi: 10.1159/000538928, 38657577 PMC11250668

[6] Rafsten L Danielsson A Sunnerhagen KS. Anxiety after stroke: a systematic review and meta-analysis. J Rehabil Med. (2018) 50:769–78. doi: 10.2340/16501977-2384, 30184240

[7] Winstein CJ Stein J Arena R Bates B Cherney LR Cramer SC . Guidelines for adult stroke rehabilitation and recovery: a guideline for healthcare professionals from the American Heart Association/American Stroke Association. Stroke. (2016) 47:e98–e169. doi: 10.1161/STR.0000000000000098, 27145936

[8] Coleman ER Moudgal R Lang K Hyacinth HI Awosika OO Kissela BM . Early rehabilitation after stroke: a narrative review. Curr Atheroscler Rep. (2017) 19:59. doi: 10.1007/s11883-017-0686-6, 29116473 PMC5802378

[9] Chollet F Cramer SC Stinear C Kappelle LJ Baron JC Weiller C . Pharmacological therapies in post stroke recovery: recommendations for future clinical trials. J Neurol. (2014) 261:1461–8. doi: 10.1007/s00415-013-7172-z, 24221642

[10] Kruse AM Worm MS Olsen KR. Social inequality in rehabilitation after stroke: a health care gap approach. Public Health. (2025) 246:105835. doi: 10.1016/j.puhe.2025.105835, 40578270

[11] Kayola G Mataa MM Asukile M Chishimba L Chomba M Mortel D . Stroke rehabilitation in low- and middle-income countries: challenges and opportunities. Am J Phys Med Rehabil. (2023) 102:S24–32. doi: 10.1097/PHM.0000000000002128, 36634327 PMC9846582

[12] He JL Ma LX Wen JS Zhuang YX Qian X Ma LH . Potential impacts of acupuncture on motor function recovery after ischemic stroke: insights from basic and clinical studies. Front Cell Neurosci. (2025) 19:1623535. doi: 10.3389/fncel.2025.1623535, 40881626 PMC12380763

[13] Sun Z Sun H Yu K Zhu Z Lin Y Sun K . A systematic review and meta-analysis of acupuncture's impact on hemiplegic gait recovery after stroke. Complement Ther Med. (2025) 91:103181. doi: 10.1016/j.ctim.2025.103181, 40288546

[14] Wang X Xiao L Lin X Zhang F Zhang K Dong B. Efficacy and safety of acupuncture or moxibustion combined with rehabilitation therapy for post-stroke shoulder-hand syndrome: a systematic review and meta-analysis of randomized controlled trials. J Stroke Cerebrovasc Dis. (2026) 35:108509. doi: 10.1016/j.jstrokecerebrovasdis.2025.108509, 41325795

[15] Hu C Wang X Pan T. Effect of acupuncture combined with lower limb gait rehabilitation robot on improving walking function in stroke patients with hemiplegia. NeuroRehabilitation. (2024) 54:309–17. doi: 10.3233/NRE-230258, 38306065

[16] Page MJ McKenzie JE Bossuyt PM Boutron I Hoffmann TC Mulrow CD . The PRISMA 2020 statement: an updated guideline for reporting systematic reviews. BMJ. (2021) 372:n71. doi: 10.1136/bmj.n71, 33782057 PMC8005924

[17] Hervé-Colas J Newton SP Engelter ST Hayward KS Held JPO Intering N . Standardized international manual of the Fugl-Meyer assessment of motor function after stroke. Neurorehabil Neural Repair. (2026) 40:306–19. doi: 10.1177/15459683251412300, 41773341 PMC13005898

[18] Wang YC Chang PF Chen YM Lee YC Huang SL Chen MH . Comparison of responsiveness of the Barthel index and modified Barthel index in patients with stroke. Disabil Rehabil. (2023) 45:1097–102. doi: 10.1080/09638288.2022.2055166, 35357990

[19] Harb A Margetis K Kishner S. "Modified Ashworth scale". In: StatPearls [Internet]. Treasure Island (FL) StatPearls Editorial Team: StatPearls Publishing: (2025) 32119459

[20] Babić A Barcot O Visković T Šarić F Kirkovski A Barun I . Frequency of use and adequacy of Cochrane risk of bias tool 2 in non-Cochrane systematic reviews published in 2020: Meta-research study. Res Synth Methods. (2024) 15:430–40. doi: 10.1002/jrsm.1695, 38262609

[21] Dong Z Li P Xu J Wang S Li Z Yang N . Effect of traditional Chinese medicine non-pharmacological interventions on cognitive function in patients with mild cognitive impairment: a systematic review and network meta-analysis. Front Med. (2026) 13:1799759. doi: 10.3389/fmed.2026.1799759, 42051696 PMC13110958

[22] Battaglia M Borg MB Loro A Cosenza L Scotti L Picelli A . Post-stroke spasticity: follow-up and functional implications of chronic long-term treatment with botulinum toxin. Eur J Phys Rehabil Med. (2024) 60:581–90. doi: 10.23736/S1973-9087.24.08429-6, 38888735 PMC11391392

[23] Wang X Xiao L Xiao L Tian C Liu Y Dai X. The dose-effect relationship of acupuncture on limb dysfunction after acute stroke: a systematic review and meta-analysis. Front Neurol. (2024) 15:1341560. doi: 10.3389/fneur.2024.1341560, 38481941 PMC10933065

[24] Hu Y Zhong D Xiao Q Chen Q Li J Jin R. Kinesio taping for balance function after stroke: a systematic review and Meta-analysis. Evid Based Complement Alternat Med. (2019) 2019:1–15. doi: 10.1155/2019/8470235, 31379969 PMC6662277

[25] Qian X Ma LX Ma LH Zhang QY Xiu JY Wang XY . Effects of acupoints-based TENS combined with tDCS on spasticity and motor function in ischemic stroke with spastic hemiplegia: study protocol for a randomized controlled trial. Front Neurol. (2023) 14:1269472. doi: 10.3389/fneur.2023.1269472, 38090263 PMC10712313

[26] Yang F. Clinical observation on treatment of spastic hemiplegia after stroke with meridian-round moxibustion. China Folk Ther. (2021) 29:34–7. doi: 10.19621/j.cnki.11-3555/r.2021.1913

[27] Huo X Zhao B Zhou Y Huang H. Effect of moxibustion combined with rehabilitation therapy on quality of life in patients with spastic hemiplegia after stroke. Liaoning J Tradit Chin Med. (2013) 40:2566–7. doi: 10.13192/j.issn.1000-1719.2013.12.070

[28] Chen Z. Effect of meridian-round moxibustion combined with reflex inhibition pattern on neurological function recovery of spastic hemiplegia after stroke. J External Ther Tradit Chin Med. (2018) 27:33–4.

[29] Xu Y Liu Y Peng S Chen Y. Curative effect observation and nursing of moxibustion in the treatment of spastic hemiplegia after stroke. China Pract Med. (2013) 8:195–6. doi: 10.14163/j.cnki.11-5547/r.2013.14.025

[30] Wei YX Zhao X Zhang BC. Synergistic effect of moxibustion and rehabilitation training in functional recovery of post-stroke spastic hemiplegia. Complement Ther Med. (2016) 26:55–60. doi: 10.1016/j.ctim.2016.02.014, 27261982

[31] Ye Q. Effect of traditional Chinese medicine moxibustion combined with rehabilitation training on limb function and activities of daily living in patients with acute cerebral infarction in recovery period. New J Tradit Chin Med. (2022) 54:203–7. doi: 10.13457/j.cnki.jncm.2022.09.045

[32] Xie L Xiaobo Y. Clinical observation of electroacupuncture combined with training to improve motor function and serum brain injury markers in patients with ischemic stroke. J Acupunct Tuina Sci. (2024) 22:127–33. doi: 10.1007/s11726-024-1428-2

[33] Zhu F Gao J Gao R He Y Liu L Ai B. Clinical efficacy of electroacupuncture combined with motor imagery therapy on hemiplegic cerebral infarction. Zhongguo Zhen Jiu. (2017) 37:927–31. doi: 10.13703/j.0255-2930.2017.09.004, 29354911

[34] Zhou L Peng L Mu J Cheng J Fang W. Effect of electroacupuncture combined with facilitation technique on walking function in patients with acute stroke hemiplegia. Shanghai J Acupunct Moxibust. (2011) 30:594–5.

[35] Fu Y Liu J Wang D Zhao Y Yang Y. Effect of electroacupuncture combined with intensive limb training on motor function of hemiplegic limbs in stroke patients. Nurs Res. (2013) 27:1743–5.

[36] Dai R Sun H Mo T Dou J Liu Z Wang Y. Effect of electroacupuncture combined with exercise therapy on limb function in patients with acute stroke. Yunnan Med J. (2016) 37:8–12. doi: 10.20282/j.cnki.1006-4141.2016.01.003

[37] Hu D Zhao M Li X. Clinical study on electroacupuncture combined with rehabilitation training in the treatment of spastic state of hemiplegia after stroke. J Acupunct Clin. (2017) 33:26–9.

[38] Zhang Y. Effect observation of electroacupuncture at antagonistic muscle acupoints combined with rehabilitation training in the treatment of spastic hemiplegia after stroke. China Pract Med. (2023) 18:153–5. doi: 10.14163/j.cnki.11-5547/r.2023.03.045

[39] Fu Q Pei J You X Gu Y Chen Y Song Y . Multi-center clinical transformation application study on the effect of electroacupuncture therapy based on yin-yang meridian syndrome differentiation on community stroke hemiplegia patients. Shanghai J Tradit Chin Med. (2016) 50:45–9. doi: 10.16305/j.1007-1334.2016.10.013

[40] Zeng H Lai Y Liu W. Clinical observation on 35 cases of spastic hemiplegia after stroke treated with fire acupuncture combined with Bobath therapy. New J Tradit Chin Med. (2015) 47:200–2. doi: 10.13457/j.cnki.jncm.2015.12.090

[41] Xiao Y Li Y Deng Y Yang T. Clinical study on treatment of spastic hemiplegia after stroke with filiform fire acupuncture combined with rehabilitation training. China Med Innov. (2016) 13:134–6.

[42] Zhang C He X Li Z Liu X. Clinical observation on treatment of hemiplegia after stroke with filiform fire acupuncture combined with comprehensive rehabilitation therapy. J Basic Tradit Chin Med. (2023) 2:83–9. doi: 10.20065/j.cnki.btcm.20230274

[43] Xu L Li F Wang M Yan X Du Y. Scalp acupuncture combined with suspension training for balance dysfunction in stroke patients: a randomized controlled study. China Acupunct Moxibust. (2021) 41:1308–12. doi: 10.13703/j.0255-2930.20201217-0002 34936265

[44] Zhao G. Yang T. Feasibility analysis of interactive scalp acupuncture in improving somatic and physiological functions of patients with hemiplegia after stroke. J Jilin Univ Tradit Chin Med (2019) 39:404–406, 420. doi: 10.13463/j.cnki.jlzyy.2019.03.036

[45] Wang J Pei J Khiati D Fu Q Cui X Song Y . Acupuncture treatment on the motor area of the scalp for motor dysfunction in patients with ischemic stroke: study protocol for a randomized controlled trial. Trials. (2017) 18:287. doi: 10.1186/s13063-017-2000-x, 28633675 PMC5479040

[46] Wang Y Zhang L. Effect of synchronous treatment with scalp acupuncture and rehabilitation training on motor dysfunction in patients with cerebral infarction. Mod J Integr Tradit Chin West Med. (2010) 19:29–30.

[47] Pang L Fu X. Clinical observation on scalp acupuncture combined with mirror therapy for hemiplegic gait after stroke. Shandong Med J. (2022) 62:76–9.

[48] Zhang W. Clinical study on scalp acupuncture combined with rehabilitation in the treatment of hemiplegia patients with cerebral thrombosis. J Yanan Univ (Med Sci Ed). (2015) 13:35–7.

[49] Xu Y Wang J Li H Yang F. Effect of scalp acupuncture combined with rehabilitation training on motor function and activities of daily living in patients with spastic stage after stroke. China Rehabil. (2015) 30:120–1.

[50] Li Z Pei J Zhan Y Kou S. Effect of scalp acupuncture combined with rehabilitation training on brain functional connectivity in patients with motor dysfunction after stroke. Chin J Rehabil Med. (2025) 40:375–81.

[51] Hu H. Value evaluation of scalp acupuncture combined with rehabilitation training in the treatment of motor dysfunction in patients with acute stroke. Asia-Pac Tradit Med. (2018) 14:153–4.

[52] Xiao L. Effect of jiao's scalp acupuncture on motor dysfunction in patients with ischemic stroke. J Jilin Univ Tradit Chin Med. (2018) 38:597–600. doi: 10.13463/j.cnki.jlzyy.2018.05.029

[53] Huang Z Huang P. Improvement effect of warm acupuncture on motor function in patients with spastic hemiplegia after stroke. Sichuan J Tradit Chin Med. (2018) 36:184–6.

[54] Yin G. Improvement effect of warm acupuncture on motor function in patients with spastic hemiplegia after stroke. Clin J Chin Med. (2021) 13:71–3.

[55] Miao L. Clinical study on warm acupuncture adjuvant treatment of spastic hemiplegia of limbs after stroke. J Pract Tradit Chin Med. (2023) 39:994–6.

[56] Hu C Li X Zhou C Liu Y Tang J Zhou M. Clinical study on warm acupuncture combined with reflex inhibition pattern in the treatment of spastic hemiplegia after stroke. New J Tradit Chin Med. (2018) 50:202–5. doi: 10.13457/j.cnki.jncm.2018.09.058

[57] Wu Y Zhang H. Effect of warm acupuncture combined with early limb rehabilitation training on rehabilitation effect of patients with hemiplegia after cerebral infarction. Chin Foreign Med Res. (2024) 3:112–4.

[58] Li F Chen J Tu M Liu S. Curative effect analysis of warm acupuncture combined with rehabilitation training in the treatment of spastic hemiplegia after stroke. J Acupunct Clin. (2019) 35:14–7.

[59] Han Z Qi L Chu L Zhou Y Zhang H Xu W . Clinical observation on the effect of Guci warm acupuncture combined with facilitation technique on motor function of spastic hemiplegia in patients with ischemic stroke. Chin J Rehabil Med. (2018) 33:1190–3.

[60] Sun H Gao X Jing R Li S. Clinical observation on the treatment of stroke hemiplegia patients with Xingnao Kaiqiao acupuncture combined with Bobath therapy. Sichuan J Tradit Chin Med. (2014) 32:146–8.

[61] Huang Y Hu L. Xingnao Kaiqiao acupuncture combined with rehabilitation training for stroke hemiplegia. Med Diet Health. (2020) 18:45–8.

[62] Wang X Gao C. Xingnao Kaiqiao acupuncture combined with rehabilitation training for stroke hemiplegia. J Hebei Med Univ. (2008) 29:559–60.

[63] Chen X. Xingnao kaiqiao acupuncture combined with rehabilitation training in the treatment of 50 cases of stroke. Chin Mod Dist Educ Tradit Chin Med. (2016) 14:97–9.

[64] Pan R Yang Z Guo Y Zhan L Zhou M Zhan J . Effect of Xingnao kaiqiao acupuncture combined with rehabilitation training on motor function and activities of daily living in patients with hemiplegia after stroke. J Guangzhou Univ Tradit Chin Med. (2015) 32:618–22. doi: 10.13359/j.cnki.gzxbtcm.2015.04.008

[65] Wang M. Effect of Xingnao kaiqiao acupuncture combined with rehabilitation treatment on nerve and limb function in patients with stroke hemiplegia. Chin Community Dr. (2020) 36:101–2.

[66] Luan H. Curative effect analysis of Xingnao kaiqiao acupuncture combined with rehabilitation training in improving hemiplegia after stroke. J Integr Tradit Chin West Med Res (2017) 9:71–72, 75.

[67] Sun K Shen W Han D Zhuang L. Effect of "Jin’s three needles" on limb motor function and activities of daily living in patients with hemiplegia after stroke. J Tradit Chin Med. (2010) 51:524–7. doi: 10.13288/j.11-2166/r.2010.06.013

[68] Lang J Zhuang L Jia C He J Zhou Z Han D . Clinical observation on the curative effect of "Jin’s three needles" combined with rehabilitation therapy in the treatment of spastic hemiplegia due to ischemic stroke. J Guangzhou Univ Tradit Chin Med. (2011) 28:369–73. doi: 10.13359/j.cnki.gzxbtcm.2011.04.012

[69] Lu Y Yang H Zhuang L Liu Y. Clinical observation on 40 cases of spastic hemiplegia after stroke treated with "Jin’s three needles" combined with rehabilitation training. J Tradit Chin Med. (2013) 54:1034–7. doi: 10.13288/j.11-2166/r.2013.12.014

[70] Li Z Chen C Yu X Chen Y. Clinical observation on "Jin’s three needles" combined with motor orthopedic therapy in the treatment of spastic hemiplegia after stroke. J Liaoning Univ Tradit Chin Med. (2019) 21:196–9. doi: 10.13194/j.issn.1673-842x.2019.11.051

[71] Zhou Z Zhuang L Xu Z Liao M Zhang B. Curative effect observation of Jin’s three needles combined with rehabilitation training in the treatment of ischemic stroke hemiplegia. Shanghai J Acupunct Moxibust. (2011) 30:214–6.

[72] Yang H Zhuang L Liu Y. Clinical study on Jin’s three needles in the treatment of spastic hemiplegia after stroke and its effect on rehabilitation training. Liaoning J Tradit Chin Med. (2013) 40:2349–51. doi: 10.13192/j.issn.1000-1719.2013.11.007

[73] Zhang N Liu G Yao Q Li W Huang Y Wang A . Effect of heat-reinforcing acupuncture combined with rehabilitation training on early motor function in patients with ischemic stroke hemiplegia: a randomized controlled study. China Acupunct Moxibust. (2010) 30:441–5. doi: 10.13703/j.0255-2930.2010.06.007 20578377

[74] Mao M Chen X Chen Y Rao P Liu J. Stage-oriented comprehensive acupuncture treatment plus rehabilitation training for apoplectic hemiplegia. J Tradit Chin Med. (2008) 28:90–3. doi: 10.1016/s0254-6272(08)60022-9, 18652112

[75] Zhang YY Li MY Ouyang G. Observation on the clinical curative effects of acupuncture for stroke hemiplegia according to muscle tension evolution rule. Acupunct Electrother Res. (2021) 46:225–37. doi: 10.3727/036012921X16207330235347

[76] Sun H. Acupuncture combined with rehabilitation training in the treatment of 48 cases of stroke hemiplegia. Henan J Tradit Chin Med. (2015) 35:537–8. doi: 10.16367/j.issn.1003-5028.2015.03.0227

[77] Chen Y Zhou H Jin T Ye T Xie W. Clinical observation of the phased acupuncture for ischemic stroke hemiplegia. Zhongguo Zhen Jiu. (2018) 38:1027–34. doi: 10.13703/j.0255-2930.2018.10.001, 30672229

[78] Chen X Chen Y Mao M Mou X Rao P Bai Y. Clinical study on acupuncture combined with modern rehabilitation technology in the treatment of spastic state of hemiplegia after stroke. J Chengdu Univ Tradit Chin Med. (2010) 33:27–9. doi: 10.13593/j.cnki.51-1501/r.2010.01.032

[79] Xia Q Zhang J Zhu D. Effect evaluation of acupuncture combined with rehabilitation training on limb function in stroke patients. World Latest Med Inform. (2019) 19:153–154, 164. doi: 10.19613/j.cnki.1671-3141.2019.32.106

[80] Wu H. Effect of acupuncture combined with rehabilitation training on improving neurological function in stroke hemiplegia patients in recovery period. Contemp Med. (2022) 28:92–5.

[81] Wang H Peng X Xu J Jia Y Min W Bian N . Effect of acupuncture combined with rehabilitation training on limb motor function and activities of daily living in stroke hemiplegia patients. Shaanxi J Tradit Chin Med. (2015) 36:914–6.

[82] Huang C. Curative effect observation of acupuncture combined with rehabilitation training on neurological function and motor disorders in patients with cerebral infarction. J Guangdong Med Coll. (2014) 32:811–2.

[83] Guo Y. Effect of acupuncture combined with rehabilitation training on activities of daily living and limb motor function in patients with hemiplegia after cerebral infarction. Sichuan J Tradit Chin Med. (2016) 34:174–7.

[84] Hao C. Curative effect study on acupuncture combined with rehabilitation training for stroke hemiplegia. China Health Stand Manag. (2019) 10:108–10.

[85] Zheng D. Clinical effect observation of traditional Chinese acupuncture combined with rehabilitation treatment on stroke hemiplegia. J Basic Med Forum. (2019) 23:1887–8. doi: 10.19435/j.1672-1721.2019.13.071

[86] Liu J. Study on the effect of acupuncture combined with modern rehabilitation therapy on vascular microenvironment in patients with sequelae of cerebral infarction. J Liaoning Univ Tradit Chin Med. (2019) 21:136–9. doi: 10.13194/j.issn.1673-842x.2019.10.036

[87] Li L Zhu Z Yu X Li Y. Clinical effect observation of acupuncture combined with rehabilitation training in the treatment of hemiplegia after stroke. Inner Mongolia J Tradit Chin Med. (2017) 36:109–10. doi: 10.16040/j.cnki.cn15-1101.2017.15.097

[88] Hu X. Observation on the improvement of stroke hemiplegia and neurological function in patients treated with traditional Chinese acupuncture combined with rehabilitation. Inner Mongolia J Tradit Chin Med. (2022) 41:72–4. doi: 10.16040/j.cnki.cn15-1101.2022.09.076

[89] Wang Q Gan Z Chen M Chen X. Clinical observation on 90 cases of hemiplegia after stroke treated with abdominal acupuncture combined with rehabilitation training. Inner Mongolia J Tradit Chin Med. (2014) 33:55–6. doi: 10.16040/j.cnki.cn15-1101.2014.18.052

[90] Jin L. Lang B. Li X. Clinical curative effect observation of abdominal acupuncture combined with exercise therapy in the treatment of spastic hemiplegia after stroke. China Mod Dr (2018) 56:94–96, 100.

[91] Wang C Li H Yu K. Effect of abdominal acupuncture combined with early rehabilitation training on motor function and cognitive impairment in patients with cerebrovascular disease. Mod J Integr Tradit Chin West Med. (2015) 24:2109–11.

[92] Wang K Sun X Luo T Zhang P Wang D Wei C. Clinical observation on eye acupuncture combined with motor function training in the treatment of ischemic stroke. J Liaoning Univ Tradit Chin Med. (2023) 25:158–62. doi: 10.13194/j.issn.1673-842x.2023.08.034

[93] Dong Y Tan L Liu J. Effect of eye acupuncture combined with ultra-early rehabilitation training on swallowing dysfunction and limb function recovery in patients with acute stroke. J Acupunct Clin. (2021) 37:9–13. doi: 10.19917/j.cnki.1005-0779.021089

[94] Sun Y Li B Hu N. Curative effect observation of eye acupuncture combined with rehabilitation training with needle retention in the treatment of limb dysfunction of hemiplegia after stroke. J Jilin Univ Tradit Chin Med. (2021) 41:272–4. doi: 10.13463/j.cnki.jlzyy.2021.02.036

[95] Zou X Wang J. Effect of eye acupuncture therapy on language and motor function in patients with stroke in rehabilitation period. J Pract Tradit Chin Intern Med. (2021) 35:108–11. doi: 10.13729/j.issn.1671-7813.z20200970

[96] Huang S. Analysis of clinical application value of floating acupuncture therapy combined with rehabilitation training in stroke hemiplegia patients. J Acupunct Clin. (2016) 32:22–4. doi: 10.19917/j.cnki.1005-0779.2016.05.008

[97] Ding LL Hu SF He XW Zhang P Zhao FF Cheng LH . Warm acupuncture therapy alleviates neuronal apoptosis after spinal cord injury via inhibition of the ERK signaling pathway. J Spinal Cord Med. (2023) 46:798–806. doi: 10.1080/10790268.2022.2088498, 35792817 PMC10446778

[98] Yang L Tan JY Ma H Zhao H Lai J Chen JX . Warm-needle moxibustion for spasticity after stroke: a systematic review of randomized controlled trials. Int J Nurs Stud. (2018) 82:129–38. doi: 10.1016/j.ijnurstu.2018.03.013, 29631145

[99] Xie J Li J Sun Q Jiang J. Multidimensional evaluation of the clinical efficacy and potential mechanisms of acupuncture combined with rehabilitation training in the treatment of stroke: a study based on multiple evaluation indicators. Front Neurol. (2025) 16:1573073. doi: 10.3389/fneur.2025.1573073, 40470494 PMC12133552

[100] Mu JD Ma LX Zhang Z Qian X Zhang QY Ma LH . The factors affecting neurogenesis after stroke and the role of acupuncture. Front Neurol. (2023) 14:1082625. doi: 10.3389/fneur.2023.1082625, 36741282 PMC9895425

[101] Min YJ Yao HH Wang L Cheng LH Hong ES. Comparison and effect of moxibustion and acupuncture on Nogo/NgR signaling pathway in rats with cerebral ischemia/reperfusion injury. J Tradit Complement Med. (2023) 13:430–40. doi: 10.1016/j.jtcme.2023.03.006, 37693099 PMC10491986

[102] Qin S Zhang Z Zhao Y Liu J Qiu J Gong Y . The impact of acupuncture on neuroplasticity after ischemic stroke: a literature review and perspectives. Front Cell Neurosci. (2022) 16:817732. doi: 10.3389/fncel.2022.817732, 36439200 PMC9685811

[103] Cao BQ Tan F Zhan J Lai PH. Mechanism underlying treatment of ischemic stroke using acupuncture: transmission and regulation. Neural Regen Res. (2021) 16:944–54. doi: 10.4103/1673-5374.297061, 33229734 PMC8178780

[104] Cao JP Du YH Jia LY Yin XM Yang LH Chen LL . Understanding the relationship between vascular smooth muscle cell function and the efficacy of acupuncture in treating cerebral ischemic stroke: a preclinical meta-analysis and systematic review. J Pain Res. (2024) 17:1693–707. doi: 10.2147/JPR.S449499, 38746535 PMC11093122

[105] Chen G Huang C Liu Y Zhang Z Qi X Shi P . Efficacy and safety of grain moxibustion in hemiplegia: a systematic review and meta-analysis protocol. Medicine (Baltimore). (2019) 98:e15215. doi: 10.1097/MD.0000000000015215, 31027068 PMC6831348

[106] Liu YF Mao HL Li YJ Zhao T Wang ZM Liu YY . Interactive scalp acupuncture for hemiplegic upper extremity motor dysfunction in patients with ischemic stroke: a randomized controlled trial. Zhongguo Zhen Jiu. (2023) 43:1109–13. doi: 10.13703/j.0255-2930.20230131-0002, 37802514

[107] Zhang QY Ma LX Wang JX Ma XL Mu JD Qian X . Electrophysiological mechanisms of motion-style scalp acupuncture for treating poststroke spasticity in rats. Zhen Ci Yan Jiu. (2023) 48:986–92. doi: 10.13702/j.1000-0607.20220920, 37879948

[108] Zhou J Zhang F. A research on scalp acupuncture for cerebral infarction. J Tradit Chin Med. (1997) 17:194–7. 10437194

[109] Zhang QY Wang JX Ma LX Zhong JS Li F Qian X . Motion-style scalp acupuncture ameliorates post-stroke muscle spasticity through cerebral blood flow augmentation and 5-HT2AR-mediated spinal KCC2 reactivation. Brain Behav. (2025) 15:e71064. doi: 10.1002/brb3.71064, 41299887 PMC12657263

[110] Yang Y. Mechanism and modern clinical application of scalp acupuncture therapy. Chin Mod Dist Educ Tradit Chin Med. (2024) 22:57–59, 75.

[111] Wei Q. Clinical observation on floating acupuncture in the treatment of limb spasm after stroke. Shanxi J Tradit Chin Med. (2025) 41:34–5. doi: 10.20002/j.issn.1000-7156.2025.03.013

[112] Zhou ZH Zhuang LX Chen ZH Lang JY Li YH Jiang GH . Post-stroke shoulder-hand syndrome treated with floating-needle therapy combined with rehabilitation training: a randomized controlled trial. Zhongguo Zhen Jiu. (2014) 34:636–40. 25233646

[113] Yu W Lu C Yang X. Research progress on acupuncture in the treatment of hemiplegic shoulder pain after stroke. Hebei J Tradit Chin Med. (2025) 47:1046–9.

[114] Yang X. Yang Z. Li S. Clinical study on floating acupuncture reperfusion in improving limb spasticity in hemiplegic patients. Syst Med (2020) 5:127–128, 147. doi: 10.19368/j.cnki.2096-1782.2020.16.127

