# Supplementary material for: Comparative effectiveness of acupuncture combined with rehabilitation for post-stroke hemiplegia: a systematic review and network meta-analysis
Source: Front Neurol. 2026 Sep 29;17:1878287. doi: 10.3389/fneur.2026.1878287 (PMC13657935; doi:10.3389/fneur.2026.1878287)
Supplement: Supplementary file 1 [file Supplementary_file_1.DOCX]

# 1  Supplementary Figures and Tables

## **1.1 Supplementary Figures**

**Supplementary Figure 1** Summary of bias risk.

**Supplementary Figure 2** Bias risk plot.

**Supplementary Figure 3** Convergence diagnostic plot for FMA

**Supplementary Figure 4** Forest plot of the NMA for FMA scores.

**Supplementary Figure 5** Convergence diagnostic plot for BI.

**Supplementary Figure 6** Forest plot of the NMA for BI scores.

**Supplementary Figure 7** Convergence diagnostic plot for MBI.

**Supplementary Figure 8** Forest plot of the NMA for MBI scores.

**Supplementary Figure 9** Convergence diagnostic plot for MAS.

**Supplementary Figure 10** Forest plot of the NMA for MAS scores.

**Supplementary Figure 11** Funnel plots for publication bias assessment.

## **1.2 Supplementary Table**

# **Supplementary** Table 1 Search strategy.

## **Supplementary Table 1.1** Search strategy of PubMed.

| # | Searches |
| --- | --- |
| 1 | "Stroke"[Mesh] |
| 2 | (((((((((((((((((((((((((((((Strokes[Title/Abstract])) OR (Cerebrovascular Accident[Title/Abstract])) OR ( Cerebrovascular Accidents [Title/Abstract])) OR ( CVA (Cerebrovascular Accident)[Title/Abstract])) OR (CVAs (Cerebrovascular Accident)[Title/Abstract])) OR (Cerebrovascular Apoplexy[Title/Abstract])) OR (Apoplexy, Cerebrovascular[Title/Abstract])) OR (Vascular Accident, Brain[Title/Abstract])) OR (Brain Vascular Accident[Title/Abstract])) OR (Brain Vascular Accidents [Title/Abstract])) OR ( Vascular Accidents, Brain[Title/Abstract])) OR (Cerebrovascular Stroke[Title/Abstract])) OR (Cerebrovascular Strokes [Title/Abstract])) OR (Stroke, Cerebrovascular[Title/Abstract])) OR (Strokes, Cerebrovascular[Title/Abstract])) OR (Apoplexy [Title/Abstract])) OR (Cerebral Stroke[Title/Abstract])) OR (Cerebral Strokes[Title/Abstract])) OR (Stroke, Cerebral [Title/Abstract])) OR (Strokes, Cerebral[Title/Abstract])) OR (Stroke, Acute[Title/Abstract])) OR (Acute Stroke[Title/Abstract])) OR (Acute Strokes[Title/Abstract])) OR (Strokes, Acute[Title/Abstract])) OR (Cerebrovascular Accident, Acute[Title/Abstract])) OR ( Acute Cerebrovascular Accident[Title/Abstract])) OR (Acute Cerebrovascular Accidents [Title/Abstract])) OR (Cerebrovascular Accidents, Acute [Title/Abstract])) |
| 3 | #1 OR #2 |
| 4 | "Hemiplegia"[Mesh] |
| 5 | (((((((((((((((((((((((((((((Hemiplegias[Title/Abstract])) OR (Hemiplegia, Transient[Title/Abstract])) OR (Hemiplegias, Transient [Title/Abstract])) OR (Transient Hemiplegia[Title/Abstract])) OR (Transient Hemiplegias[Title/Abstract])) OR (Monoplegia[Title/Abstract])) OR (Monoplegias[Title/Abstract])) OR (Hemiplegia, Post-Ictal[Title/Abstract])) OR (Hemiplegia, Post Ictal[Title/Abstract])) OR (Hemiplegias, Post-Ictal [Title/Abstract])) OR (Post-Ictal Hemiplegia[Title/Abstract])) OR ( Post-Ictal Hemiplegias[Title/Abstract])) OR (Hemiplegia, Crossed[Title/Abstract])) OR (Crossed Hemiplegia[Title/Abstract])) OR (Crossed Hemiplegias[Title/Abstract])) OR (Hemiplegias, Crossed[Title/Abstract])) OR (Hemiplegia,Flaccid[Title/Abstract])) OR (Flaccid Hemiplegia[Title/Abstract])) OR (Flaccid Hemiplegias[Title/Abstract])) OR (Hemiplegias, Flaccid[Title/Abstract])) OR (Hemiplegia, Infantile[Title/Abstract])) OR (Hemiplegias, Infantile [Title/Abstract])) OR (Infantile Hemiplegia[Title/Abstract])) OR (Infantile Hemiplegias[Title/Abstract])) OR (Hemiplegia, Spastic[Title/Abstract])) OR (Hemiplegias, Spastic[Title/Abstract])) OR (Spastic Hemiplegia[Title/Abstract])) OR (Spastic Hemiplegias [Title/Abstract])) |
| 6 | #4 OR #5 |
| 7 | "Acupuncture"[Mesh] |
| 8 | ((((((((Electroacupuncture[Title/Abstract])) OR (Xingnao Kaiqiao Acupuncture [Title/Abstract])) OR (Moxibustion [Title/Abstract])) OR (Needle [Title/Abstract])) OR (Scalp Acupuncture[Title/Abstract])) OR (Fire needle[Title/Abstract])) OR (warm needle moxibustion[Title/Abstract])) |
| 9 | #7 OR #8 |
| 10 | "Acupuncture"[Mesh] |
| 11 | ((((practice[Title/Abstract])) OR ( exercise [Title/Abstract])) OR (train[Title/Abstract])) |
| 12 | #10 OR #11 |
| 13 | ((((randomized controlled trial[Publication Type])) OR (randomized[Title/Abstract])) OR (placebo[Title/Abstract])) OR (RCT[Title/Abstract]) |
| 14 | #3 AND #6 AND #9 AND #12 AND #13 |

## **Supplementary Table 1.2** Search strategy of Web of Science.

| # | Searches |
| --- | --- |
| 1 | (((((((((((((((((((((((((((TS=(Strokes)) OR TS=( Cerebrovascular Accident )) OR TS=( Cerebrovascular Accidents )) OR TS=( CVA (Cerebrovascular Accident))) OR TS=(CVAs (Cerebrovascular Accident) )) OR TS=(Cerebrovascular Apoplexy )) OR TS=( Apoplexy, Cerebrovascular)) OR TS=(Vascular Accident, Brain)) OR TS=(Brain Vascular Accident)) OR TS=(Brain Vascular Accidents )) OR TS=(Vascular Accidents, Brain)) OR TS=(Cerebrovascular Stroke)) OR TS=(Cerebrovascular Strokes)) OR TS=(Stroke, Cerebrovascular)) OR TS=(Strokes, Cerebrovascular)) OR TS=(Apoplexy)) OR TS=( Cerebral Stroke)) OR TS=(Cerebral Strokes)) OR TS=(Stroke, Cerebral)) OR TS=(Strokes, Cerebral)) OR TS=(Stroke, Acute)) OR TS=(Acute Stroke)) OR TS=(Acute Strokes )) OR TS=(Strokes, Acute )) OR TS=(Cerebrovascular Accident, Acute)) OR TS=(Acute Cerebrovascular Accident)) OR TS=(Acute Cerebrovascular Accidents )) OR TS=(Cerebrovascular Accidents, Acute ) |
| 2 | ((((((((((((((((((((((((((((TS=(hemiplegia )) OR TS=(HemiplegiasHemiplegia, Transient)) OR TS=(Hemiplegias )) OR TS=(Hemiplegias, Transient )) OR TS=(Transient Hemiplegia )) OR TS=(Transient Hemiplegias)) OR TS=(Monoplegia)) OR TS=(Monoplegias)) OR TS=(Hemiplegia, Post-Ictal)) OR TS=(Hemiplegia, Post Ictal)) OR TS=(Hemiplegias, Post-Ictal )) OR TS=(Post-Ictal Hemiplegia)) OR TS=(Post-Ictal Hemiplegias)) OR TS=(Hemiplegia, Crossed)) OR TS=(Crossed Hemiplegia)) OR TS=(Crossed Hemiplegias)) OR TS=(Hemiplegias, Crossed)) OR TS=(Hemiplegia, Flaccid)) OR TS=(Flaccid Hemiplegia)) OR TS=(Flaccid Hemiplegias)) OR TS=(Hemiplegias, Flaccid )) OR TS=(Hemiplegia, Infantile )) OR TS=(Hemiplegias, Infantile)) OR TS=(Infantile Hemiplegia)) OR TS=(Infantile Hemiplegias)) OR TS=(Hemiplegia, Spastic)) OR TS=(Hemiplegias, Spastic)) OR TS=(Spastic Hemiplegia)) OR TS=(Spastic Hemiplegias ) |
| 3 | (((((((TS=(Acupuncture)) OR TS=(Electroacupuncture)) OR TS=(Xingnao Kaiqiao Acupuncture)) OR TS=(Needle)) OR TS=(Scalp Acupuncture)) OR TS=(Fire needle)) OR TS=(warm needle moxibustion)) OR TS=(Moxibustion ) |
| 4 | (((TS=(Rehabilitation)) OR TS=(practice)) OR TS=(exercise)) OR TS=(train) |
| 5 | ((((TS=(RCT)) OR TS=(randomized clinical trials)) OR TS=(randomized controlled clinical trial)) OR TS=(randomized controlled trial)) OR TS=(randomized experimen) |
| 6 | #1 AND #2 AND #3 AND #4 AND #5 |

## **Supplementary Table 1.3** Search strategy of Cochrane Library.

| # | Searches |
| --- | --- |
| 1 | MeSH descriptor: [Stroke] explode all trees |
| 2 | (Cerebrovascular Accidents, Acute):ti,ab,kw OR (Acute Stroke):ti,ab,kw OR (Acute Cerebrovascular Accident):ti,ab,kw OR (Acute Strokes):ti,ab,kw OR (Stroke, Acute):ti,ab,kw |
| 3 | #1 OR #2 |
| 4 | MeSH descriptor: [Cerebral Infarction] explode all trees |
| 5 | (Cerebrovascular Accident, Acute):ti,ab,kw OR (Brain Vascular Accidents):ti,ab,kw OR (Cerebrovascular Accidents):ti,ab,kw OR (Cerebral Strokes):ti,ab,kw OR (Vascular Accident, Brain):ti,ab,kw |
| 6 | #4 OR #5 |
| 7 | #3 OR #6 |
| 8 | MeSH descriptor: [Hemiplegia] explode all trees |
| 9 | (Crossed Hemiplegia):ti,ab,kw OR (Hemiplegia, Crossed):ti,ab,kw OR (Hemiplegia, Spastic):ti,ab,kw OR (Flaccid Hemiplegia):ti,ab,kw OR (Hemiplegias, Infantile):ti,ab,kw |
| 10 | #8 or #9 |
| 11 | MeSH descriptor: [Hemiplegia] explode all trees |
| 12 | (Hemiplegias, Infantile):ti,ab,kw OR (Infantile Hemiplegias):ti,ab,kw OR (Hemiplegia, Infantile):ti,ab,kw OR (Infantile Hemiplegia):ti,ab,kw OR (Hemiplegias, Post-Ictal):ti,ab,kw |
| 13 | #11 OR #12 |
| 14 | #10 OR #13 |
| 15 | MeSH descriptor: [Acupuncture] explode all trees |
| 16 | (Pharmacopuncture):ti,ab,kw OR (Electroacupuncture):ti,ab,kw OR (Xingnao Kaiqiao Acupuncture):ti,ab,kw OR (Moxibustion):ti,ab,kw OR (Needle):ti,ab,kw |
| 17 | #15 OR #16 |
| 18 | MeSH descriptor: [Rehabilitation] explode all trees |
| 19 | (practice):ti,ab,kw OR (exercise):ti,ab,kw OR (train):ti,ab,kw |
| 20 | #18 OR #19 |
| 21 | #7 AND #14 AND #17 #20 |

## **Supplementary Table 1.4** Search strategy of Embase.

| # | Searches |
| --- | --- |
| 1 | 'cerebrovascular accident'/exp |
| 2 | 'accident, cerebrovascular':ti,ab,kw OR 'acute cerebrovascular lesion':ti,ab,kw OR 'acute focal cerebral vasculopathy':ti,ab,kw OR 'acute stroke':ti,ab,kw OR 'apoplectic stroke':ti,ab,kw OR 'apoplexia':ti,ab,kw OR apoplexy:ti,ab,kw OR 'blood flow disturbance, brain':ti,ab,kw OR 'brain accident':ti,ab,kw OR 'brain attack':ti,ab,kw OR 'brain blood flow disturbance':ti,ab,kw OR 'brain insult':ti,ab,kw OR 'brain insultus':ti,ab,kw OR 'brain vascular accident':ti,ab,kw OR 'cerebral apoplexia':ti,ab,kw OR 'cerebral insult':ti,ab,kw OR 'cerebral stroke':ti,ab,kw OR 'cerebral vascular accident':ti,ab,kw OR 'cerebral vascular insufficiency':ti,ab,kw OR 'cerebro vascular accident':ti,ab,kw OR 'cerebrovascular arrest':ti,ab,kw OR 'cerebrovascular failure':ti,ab,kw OR 'cerebrovascular injury':ti,ab,kw OR 'cerebrovascular insufficiency':ti,ab,kw OR 'cerebrovascular insult':ti,ab,kw OR 'cerebrum vascular accident':ti,ab,kw OR 'cryptogenic stroke':ti,ab,kw OR cva:ti,ab,kw OR 'insultus cerebralis':ti,ab,kw OR 'ischaemic seizure':ti,ab,kw OR 'ischemic seizure':ti,ab,kw OR 'stroke':ti,ab,kw OR 'thrombotic stroke':ti,ab,kw OR 'cerebrovascular accident':ti,ab,kw |
| 3 | #1 OR #2 |
| 4 | 'hemiplegia'/exp |
| 5 | 'hemiplegia':ti,ab,kw OR 'hemiparalysis':ti,ab,kw OR 'hemiplegic':ti,ab,kw OR 'hemiplegy':ti,ab,kw |
| 6 | #4 OR #5 |
| 7 | 'acupuncture'/exp |
| 8 | 'acupuncture':ti,ab,kw OR 'acupuncture therapy':ti,ab,kw OR 'shonishin':ti,ab,kw OR needle:ti,ab,kw OR moxibustion:ti,ab,kw |
| 9 | #7 OR #8 |
| 10 | 'Rehabilitation'/exp |
| 11 | rehabilitation:ti,ab,kw OR 'functional readaptatio':ti,ab,kw OR 'medical rehabilitation':ti,ab,kw OR readaption:ti,ab,kw OR 'rehabilitation concept':ti,ab,kw OR 'rehabilitation engineering':ti,ab,kw OR 'rehabilitation potential':ti,ab,kw OR 'rehabilitation process':ti,ab,kw OR 'rehabilitation program':ti,ab,kw OR 'rehabilitation programme':ti,ab,kw OR 'rehabilitation, medical':ti,ab,kw OR 'rehabilitational program':ti,ab,kw OR 'rehabilitational programme':ti,ab,kw OR 'rehabilitational treatment':ti,ab,kw OR resocialisation:ti,ab,kw OR 'resocialisation therapy':ti,ab,kw OR 'resocialization':ti,ab,kw OR 'resocialization therapy':ti,ab,kw OR revalidation:ti,ab,kw |
| 12 | #10 OR #11 |
| 13 | 'randomized controlled trial'/exp |
| 14 | 'randomized controlled trial':ti,ab,kw OR placebo:ti,ab,kw OR randomised:ti,ab,kw |
| 15 | #13 OR #14 |
| 16 | #3 AND #6 AND #9 AND #12 AND #15 |

## **Supplementary Table 1.5** Search strategy of CNKI.

| # | Searches |
| --- | --- |
| 1 | （主题：针灸）OR（主题：针刺）OR（主题：电针）OR（主题：头针）OR（主题：醒脑开窍针法）OR（主题：体针）OR（主题：针）OR（主题：灸）AND（主题：康复）OR（主题：康复训练）OR（主题：运动康复）OR（主题：Bobath）OR（主题：Brunnstrom）AND（主题：脑梗死）OR（主题：中风）OR（主题：卒中）OR（主题：缺血性脑卒中）OR（主题：脑梗塞）OR（主题：脑栓塞）OR（主题：脑血栓）OR（主题：脑血管病）OR（主题：脑血管意外）OR（主题：缺血性中风）AND（主题：偏瘫）OR（主题：半身不遂）OR（主题：运动功能障碍）OR（主题：肢体功能障碍）AND（篇关摘：随机对照(精确)）OR（篇关摘：随机(精确)）OR（篇关摘：RCT(精确)） |

## **Supplementary Table 1.6** Search strategy of Wanfang.

| # | Searches |
| --- | --- |
| 1 | 题名或关键词:(脑梗死 OR 中风 OR 卒中 OR 缺血性脑卒中 OR 脑梗塞 OR 脑栓塞 OR 脑血栓 OR 脑血管病 OR 脑血管意外 OR 缺血性中风) and 题名或关键词:(偏瘫 OR 半身不遂 OR 运动功能障碍 OR 肢体功能障碍) and 题名或关键词:(针灸 OR 针刺 OR 电针 OR 头针 OR 醒脑开窍针法 OR 体针 OR 针 OR 灸) and 题名或关键词:(康复 OR 康复训练 OR 运动康复 OR Bobath OR Brunnstrom) and 摘要:(RCT OR 随机对照试验 OR 随机) |

## **Supplementary Table 1.7** Search strategy of VIP.

| # | Searches |
| --- | --- |
| 1 | (((((((((((((((((((((题名或关键词=脑梗死 OR 题名或关键词=中风) OR 题名或关键词=卒中) OR 题名或关键词=缺血性脑卒中) OR 题名或关键词=脑梗塞) OR 题名或关键词=脑栓塞) OR 题名或关键词=脑血栓) OR 题名或关键词=脑血管病) OR 题名或关键词=脑血管意外) OR 题名或关键词=缺血性中风) AND (((题名或关键词=偏瘫 OR 题名或关键词=半身不遂) OR 题名或关键词=运动功能障碍) OR 题名或关键 词=肢体功能障碍)))) AND (((((((题名或关键词=针灸 OR 题名或关键词=针刺) OR 题名或关键词=电针) OR 题名或关键词=头针) OR 题名或 关键词=醒脑开窍针法) OR 题名或关键词=体针) OR 题名或关键词=针) OR 题名或关键词=灸)))) AND ((((题名或关键词=康复 OR 题名或关 键词=康复训练) OR 题名或关键词=运动康复) OR 题名或关键词=Bobath) OR 题名或关键词=Brunnstrom)))) AND (((((((((摘要=RCT OR 摘要 =随机对照试验) OR 摘要=randomized clinical trials) OR 摘要=randomized controlled clinical trial) OR 摘要=randomized controlled trial) OR 摘要 =randomized controlled trials) OR 摘要=randomized experiment) OR 摘要=rct) OR 摘要=随机对照实验) OR 摘要=随机对照研究)))) |

## **Supplementary Table 1.8** Search strategy of CBM.

| # | Searches |
| --- | --- |
| 1 | ("随机对照试验"[常用字段:智能] OR( "RCT"[常用字段:智能] OR "randomized clinical trials"[常用字段:智能] OR "randomized controlled clinical trial"[常用字段:智能] OR "randomized controlled trial"[常用字段:智能] OR "randomized controlled trials"[常用字段:智能] OR "randomized experiment"[常用字段:智能] OR "rct"[常用字段:智能] OR "随机对照实验"[常用字段:智能] OR "随机对照研究"[常用字段:智能])) AND ("康复"[常用字段:智能] OR( "康复训练"[常用字段:智能] OR "运动康复"[常用字段:智能] OR " Bobath "[常用字段:智能] OR " Brunnstrom"[常用字段:智能])) AND ("针灸"[常用字段:智能] OR( "针刺"[常用字段:智能] OR "电针"[常用字段:智能] OR "头针"[常用字段:智能] OR "醒脑开窍针法"[常用字段:智能] OR "体针"[常用字段:智能] OR "针"[常用字段:智能] OR "灸"[常用字段:智能])) AND ("偏瘫"[常用字段:智能] OR( "半身不遂"[常用字段:智能] OR "运动功能障碍"[常用字段:智能] OR "肢体功能障碍"[常用字段:智能])) AND ("中风"[常用字段:智能] OR( "脑梗死"[常用字段:智能] OR "卒中"[常用字段:智能] OR "缺血性脑卒中"[常用字段:智能] OR "脑梗塞"[常用字段:智能] OR "脑栓塞"[常用字段:智能] OR "脑血栓"[常用字段:智能] OR "脑血管病"[常用字段:智能] OR "脑血管意外"[常用字段:智能] OR "缺血性中风"[常用字段:智能])) |

**Supplementary Table 2** Participant Attrition and Reasons for Withdrawal

| Study | Dropouts and Reasons |
| --- | --- |
| Qinhui Fu 2016 | A total of 8 participants dropped out, and the reasons were not specified. |
| Lei Xu 2021 | Two participants dropped out from each group (the intervention and control groups) due to personal or family reasons. |
| Jun Wang 2017 | In the intervention group, 3 participants were lost to follow-up. In the control group, 2 participants dropped out: one withdrew due to acute coronary syndrome, and the other was lost to follow-up due to recurrent cerebral infarction. |
| Yingying Zhang 2021 | Three participants dropped out from the observation group and 2 from the control group, with no reasons provided. |

## **Supplementary Table 3** Definition of intervention measures and control group.

| Interventions | Description |
| --- | --- |
| Moxibustion  + Rehabilitation | Moxibustion therapy (using moxa sticks or moxa pillars to generate mild thermal stimulation on acupoints or affected limbs) combined with standardized conventional rehabilitation. The treatment frequency and duration are consistent with the control group, aiming to warm meridians, promote blood circulation, and improve motor function and spasticity in post-stroke hemiplegic patients. |
| Electroacupuncture  + Rehabilitation | Electroacupuncture (EA) involves inserting filiform needles into acupoints, followed by delivery of low-intensity, low-frequency electrical current through the needles to enhance acupoint stimulation. This intervention is combined with conventional rehabilitation, with uniform treatment parameters across studies, to facilitate recovery of limb motor function and relieve spasticity. |
| Fire needle  + Rehabilitation | Fire needle therapy (rapid insertion of pre-heated thick needles into specific acupoints or affected muscle groups) combined with conventional rehabilitation. The thermal and mechanical stimulation of fire needles is applied to dredge meridians, relieve pain, and alleviate limb spasticity in post-stroke hemiplegic patients, with consistent treatment protocols. |
| Scalp acupuncture  + Rehabilitation | Scalp acupuncture (needling specific scalp regions corresponding to the motor or sensory cortex) combined with conventional rehabilitation. This technique modulates cortical excitability to promote functional recovery of hemiplegic limbs, with identical rehabilitation regimens and treatment duration as the control group. |
| Warm needle moxibustion  + Rehabilitation | Warm needle moxibustion (filiform needle insertion combined with moxa combustion on the needle handle to conduct heat into acupoints) combined with conventional rehabilitation. This integrated intervention delivers both mechanical needle stimulation and thermal moxibustion effects to improve motor function and reduce muscle spasticity. |
| Acupuncture  + Rehabilitation | Conventional acupuncture (filiform needle insertion into acupoints based on meridian or syndrome differentiation, with manipulation techniques such as twirling and lifting) combined with conventional rehabilitation. The treatment frequency and duration are consistent with the control group to regulate qi and blood circulation and improve motor function. |
| Abdominal Acupuncture  + Rehabilitation | Abdominal acupuncture (needling specific abdominal points such as Zhongwan, Qihai, and Guanyuan, with varying needling depths corresponding to different therapeutic layers) combined with conventional rehabilitation. This technique regulates visceral function and the body's innate meridian system to harmonize qi and blood, thereby promoting functional recovery, with identical rehabilitation regimens and treatment duration as the control group. |
| Eye Acupuncture  + Rehabilitation | Eye acupuncture (needling specific zones on the orbital orbit corresponding to the Liver, Kidney, Upper Jiao, and Lower Jiao) combined with ultra-early rehabilitation training. This technique stimulates the meridians around the eyes to regulate the qi and blood of internal organs, thereby improving dysphagia and limb motor function, with identical rehabilitation regimens and treatment duration as the control group. |
| Floating Acupuncture  + Rehabilitation | Floating Acupuncture (superficial needling in the subcutaneous layer at a distance of 6–10 cm from the lesion, combined with sector-shaped mobilization and reperfusion movements) combined with conventional rehabilitation. This technique differs from traditional acupuncture by avoiding deep penetration and instead focuses on loosening the subcutaneous loose connective tissue to relieve muscle spasm and pain immediately, thereby enhancing motor recovery, with identical rehabilitation regimens and treatment duration as the control group. |

**Supplementary Table 4** Egger's Test Results for Publication Bias

|  | FMA | BI | MBI |
| --- | --- | --- | --- |
| *P*-value | 0.2196 | 0.1793 | 0.3765 |
| *t*-value | 1.24 | 1.38 | -0.9 |
| degrees of freedom (df) | 60 | 27 | 21 |
| Bias estimate | 1.1748 | 1.5098 | -1.0805 |
